# 3D Profile-Based Approach to Proteome-Wide Discovery of Novel Human Chemokines

**DOI:** 10.1371/journal.pone.0036151

**Published:** 2012-05-07

**Authors:** Aurelie Tomczak, Jana Sontheimer, David Drechsel, Rainer Hausdorf, Marc Gentzel, Andrej Shevchenko, Stefanie Eichler, Karim Fahmy, Frank Buchholz, M. Teresa Pisabarro

**Affiliations:** 1 Structural Bioinformatics, BIOTEC TU Dresden, Germany; 2 Max Planck Institute of Molecular Cell Biology and Genetics, Dresden, Germany; 3 Helmholtz-Zentrum Dresden-Rossendorf, Dresden, Germany; 4 Department of Medical Systems Biology, TU Dresden, University Hospital and Medical Faculty Carl Gustav Carus, Dresden, Germany; Charité-Universitätsmedizin Berlin, Germany

## Abstract

Chemokines are small secreted proteins with important roles in immune responses. They consist of a conserved three-dimensional (3D) structure, so-called IL8-like chemokine fold, which is supported by disulfide bridges characteristic of this protein family. Sequence- and profile-based computational methods have been proficient in discovering novel chemokines by making use of their sequence-conserved cysteine patterns. However, it has been recently shown that some chemokines escaped annotation by these methods due to low sequence similarity to known chemokines and to different arrangement of cysteines in sequence and in 3D. Innovative methods overcoming the limitations of current techniques may allow the discovery of new remote homologs in the still functionally uncharacterized fraction of the human genome. We report a novel computational approach for proteome-wide identification of remote homologs of the chemokine family that uses fold recognition techniques in combination with a scaffold-based automatic mapping of disulfide bonds to define a 3D profile of the chemokine protein family. By applying our methodology to all currently uncharacterized human protein sequences, we have discovered two novel proteins that, without having significant sequence similarity to known chemokines or characteristic cysteine patterns, show strong structural resemblance to known anti-HIV chemokines. Detailed computational analysis and experimental structural investigations based on mass spectrometry and circular dichroism support our structural predictions and highlight several other chemokine-like features. The results obtained support their functional annotation as putative novel chemokines and encourage further experimental characterization. The identification of remote homologs of human chemokines may provide new insights into the molecular mechanisms causing pathologies such as cancer or AIDS, and may contribute to the development of novel treatments. Besides, the genome-wide applicability of our methodology based on 3D protein family profiles may open up new possibilities for improving and accelerating protein function annotation processes.

## Introduction

The fraction of uncharacterized proteins in the human proteome currently still comprises more than 75% in the UniProt knowledgebase, which is the most comprehensive available protein sequence database (all proteins in UniProt/TrEMBL) [1]. Current techniques to predict the function of uncharacterized proteins rely mainly on their sequence homology to already characterized proteins [2]. More advanced methods use sequence-profile [3] or profile-profile alignments [4]. Although these methods have significantly improved in terms of detection of remote homology [5], still more than 20% of the human proteome has not been annotated (*i.e.* no InterPro domain annotation in UniProt) because no characterized homologs could be identified with the necessary statistical significance.

Protein structural homology is often strong in the absence of significant sequence homology [6]. Structure-based fold recognition methods have been shown to be useful to recognize possible structural resemblances even at levels of non-recognizable sequence similarity (below 20–30% [7]). Threading techniques are able to predict the 3D structure of a query protein by pulling its amino acid sequence through the backbone of experimentally determined protein 3D structures without relying on sequence similarity. Thus, these techniques can complement sequence-based methods in structural and functional annotation of proteins. Detection of very remote homology by using these techniques has been successfully demonstrated in previous applications [8]–[11]. In our previous work, we discovered a novel remote member of the chemokine family by applying fold recognition methods, the human chemokine CXCL17 [12], which is the last member of the CXC chemokine family being so far identified [13]. Chemokines are secreted signal proteins with significant impact on the function of the immune system and are important molecules in inflammatory responses. Some chemokines have also been shown to play a role in processes like angiogenesis [14], haematopoiesis [15], inhibition of HIV infection, tumor growth [16], [17] and apoptosis [18]. Furthermore, they are very well suited for the development of small molecule inhibitors with strong therapeutic potential as they act through G protein–coupled receptors [19].

Chemokines share a conserved 3D structure, the so-called IL8-like chemokine fold, which is stabilized by cysteine residues forming intra-molecular disulfide bonds. Interestingly, the predicted IL8-like chemokine structure of CXCL17 revealed disulfide bonds in non-canonical regions in 3D structure but still maintaining an active fold. The low sequence similarity to other known members of the family and its cysteine patterns differing from those in known chemokines are the reasons why chemokine CXCL17 escaped annotation by standard sequence-based methods [12]. Current standard techniques that can be applied to identify chemokines include the ChemoPred web server [20], a machine learning technique, Hidden Markov Models of SMART [21], [22] and Pfam [23] and PROSITE’s chemokine profiles and patterns [24] (Supporting Text S1 I and Table S1). However, the results that can be obtained by these methods are limited to the diversity of the training dataset, which originates almost exclusively from sequence-based approaches. Thus, these methods can hardly detect structural resemblance without detectable sequence similarity.

The identification of CXCL17 by threading-based computational means motivated us to perform a more systematic search in the human proteome to discover other possible remote members of the chemokine family that, like CXCL17, might have been so far overlooked. Several threading techniques are available to detect structural resemblances in proteins [5], including the methodology we used to discover CXCL17 [12]. However, none of these resources offers the possibility to automatically screen thousands of sequences for structural resemblances in combination with scaffold-based mapping of disulfide bonds patterns that may be indicative of a functional fold in 3D.

Here, we present a novel computational approach based on 3D protein family profiles for searching in a proteome-wide automatic fashion for still uncharacterized human proteins that could fold into a functional IL8-like chemokine architecture and that may have not been yet discovered because of having remote sequence similarity to known chemokines and a different cysteine pattern in sequence and in 3D. We combine threading techniques for structure prediction with an automatic method for scaffold-based mapping of disulfide bonds in 3D as a functional descriptor. We apply our methodology to all currently uncharacterized human protein sequences from the UniProt database, and we identify two novel human proteins that present a strong structural resemblance to other known chemokines, in particular two anti-HIV chemokines. We build 3D molecular models of these proteins and perform detailed structure-based computational analysis in combination with experimental work based on mass spectrometry and circular dichroism, which support our structural predictions and highlight several other chemokine-like features. The results obtained substantiate the annotation of these two novel proteins as putative human chemokines and should awaken great interest in their further experimental characterization.

## Results and Discussion

**Figure 1:**
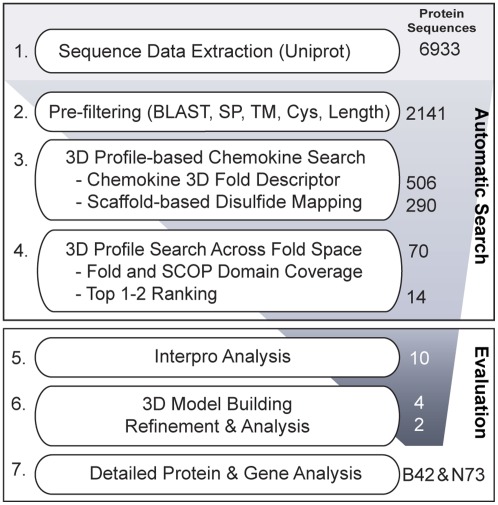
Overview of our methodology. Uncharacterized human proteins are extracted from the UniProt Knowledgebase. Steps 1–4 are fully automated. The number of proteins that remain after each filter step is summarized on the right.

The general scheme of our computational approach and the steps followed in our work to discover novel human protein sequences that could potentially be remote chemokine homologs are summarized in Figure 1.

### Proteome-wide 3D Profile-based Computational Screening

#### Sequence data extraction and pre-filtering

A search in UniProt for human protein sequences still uncharacterized was performed with the criteria of selecting sequences containing at least two cysteine residues, as the formation of disulfide bonds is a requirement for a functional IL8-like chemokine fold (for details see Materials and Methods section 1). This search resulted in a total of 6,933 protein sequences (Figure 1, step 1), which were taken as input dataset for our automatic analysis. Next, and in order to discard from our studies those sequences that could be already structurally annotated, we performed a BLAST search against the Protein Data Bank (PDB). Also signal peptides and transmembrane regions were identified and removed, as these are not included in the chemokine fold. Then, the cysteine content of the remaining sequences was again evaluated to make sure that the selected sequences would contain at least 2 cysteine residues (for details see Materials and Methods section 2). This pre-filtering resulted in a total of 2,141 uncharacterized sequences (Figure 1, step 2).

#### 3D fold family profiling

Thereafter, we generated a 3D descriptor of the architecture of the chemokine fold family (IL8-like) by using available information on 270 experimentally known chemokine structures (*Chemokine fold library*). We performed automatic 3D profiling on the pre-filtered 2,141 uncharacterized sequences by carrying out threading calculations with ProHit (ProCeryon Biosciences, ProHit Professional V 2.2.2) [25], [26] using the *Chemokine fold library* combined with an automatic scaffold-based mapping of disulfide bonds (for details see “Chemokine 3D fold descriptor” and “Automatic scaffold-based disulphide mapping” in Materials and Methods section 3). Applying our scoring and ranking criteria, we selected: i) high scoring sequences best fitting into the chemokine fold, and ii) sequences that could potentially form disulfide bonds into the predicted scaffold as a premise for being able to form a functional fold. This automatic 3D protein fold family profiling resulted on a total of 290 human protein sequences, until now uncharacterized, that could potentially fold into a functional IL8-like chemokine framework.

#### 3D profiling across fold space

Next, we performed a 3D profiling across fold space to investigate whether the best scoring of the 290 filtered uncharacterized sequences would best fit into a chemokine-like structure when compared to all other possible structures in the fold universe. For this, we performed threading control experiments across the currently available protein fold space with our 70 top scoring sequences as query. A fold library containing ∼24,000 templates representing all the three-dimensional structures currently available in the PDB (named hereafter *pdb95 fold library*) was used for these purposes. Each of the 70 query sequences was analysed for folding preferences across fold space. After applying our scoring and ranking criteria to the results obtained, those query sequences with a chemokine structure in the top 1 or 2 hits were automatically selected (for details see Materials and Methods section 4). This resulted in a total of 14 until now uncharacterized human protein sequences that showed a stronger preference to fold into a functional chemokine-like structure than into any other protein architecture known up to date, and therefore with a strong potential to be possible remote structural homologs of the IL8-like chemokine fold family.

Our fully automated 3D profile-based methodology allowed us to screen several thousands of so far uncharacterized human protein sequences to discover a few candidates, which fit our 3D chemokine fold family profile with high confidence. Further detailed analysis of these 14 high confidence protein sequences was performed in order to investigate their discovered strong structural resemblance to the IL8-like chemokine fold and to substantiate further structure-based functional predictions. The results obtained in this 3D profiling across fold space for the best two candidates (B42 and N73) are described in detail in the following sections.

### Discovery of Two Putative Remote Homologs of the IL8-like Chemokine Fold

Each of the 14 automatically selected protein sequences was separately examined for further characterization. Although the sequences of our initial dataset were selected because of being labelled as “unknown”, “hypothetical” etc. in the UniProt Knowledgebase (Materials and Methods section 1), we checked for available information that could support or exclude their annotation as potential novel chemokines. For this purpose we used the InterPro database [27], which integrates predictive models or signatures representing protein domains, families and functional sites from diverse source databases (Materials and methods section 5). Based on the InterPro signatures for each of these 14 query sequences, we discarded four of them because they refuted a chemokine-like fold: two were listed as transmembrane, one as nucleotide-binding, and another one as a zinc-finger protein. The high confidence sequence-to-structure alignments obtained from our threading calculations with the *pdb95 fold library* (*i.e.* chemokine fold in the top 1 or 2 hits; see above) were used to generate atomic 3D models of each of the resulting 10 query protein sequences using their corresponding top 1 or 2 ranking chemokine fold as structural template (see Materials and Methods section 6 and Table S2). The obtained 3D models were energy minimized by molecular dynamics and, upon analysis of the results (for details see “Analysis of models in Materials and Methods section 6), six models were discarded because they did not show the compact packing expected for a properly folded structure (data not shown).

As a quality control and in order to compare the remaining four 3D models, (proteins G19, L32, B42 and N73; see Table 1 and Table S2) with models of known chemokine structures, we built as reference 3D models of the proteins vMIP-I and vMIP-II used as templates to model our query proteins, and a model of the remote member of the chemokine family CXCL17 [12] (see “Reference models” in Materials and Methods section 6). These control models were refined with the same MD protocol applied to the query protein models. After evaluation of the MD results using the control models as reference and taking into account overall structure, packing of the protein core and contact residue energetics (see “Analysis of models” in Materials and Methods section 6), the models of proteins G19 and L32 were considered of low quality because they did not show agreement with their respective reference models (data not shown). The models of proteins B42 and N73 showed good quality in agreement with their respective controls, and were therefore considered as high confidence predictions of chemokine-like structures. Based on our results we hence predicted proteins B42 and N73 to be high confidence structural homologs of the chemokine fold family.

**Table 1:** Summary of sequence and structural properties of the best four predictions and chemokine CXCL17 as reference.

Filter Step:		1) Sequence Data Extraction	2) Pre-filtering		3) 3D Fold Family Profiling				4) 3D Profiling Across Fold Space	6) Modelling & Analysis	7) Protein & gene analysis					
ID	Conf	UniProt	Seq	Cys	Thx	%ID	CK Template	diS	CK Rank	Control	SP	secP	Loc	Exon	Iph	ChrLoc
CXCL17	TP	Q6UXB2	24–109	6	25.3	14.3	CXCL8	2	4	+	+	−	Ex	4	111	19q13.2
B42	HC	Q1T7F1	1–81	6	45.6	24.2	vMIP-IA	2–3	1	+	−	+	Ex	3	2	19q13.41
N73	HC	Q71RG6	53–208	8	36.3	23.2	vMIP-II	2–3	2	+	+	−	Ex	3	−	8q24.3
G19	LC	Q96KT8	1–98	7	45.4	22.9	vMIP-II	2	1	−	−	−	Ex	1	−	8p23.1
L32	LC	Q8N9X3	1–169	7	33.2	21.9	CCL14	1	2	−	−	+	Nu	3,4,7	−	1p13.2

*ID*: Protein name; *Conf*: confidence level of prediction (TP, true positive; HC, high confidence; LC, low confidence); *UniProt*: UniProt identifier; *Seq*: sequence region selected; *Cys*: number of cysteines remaining after the pre-filtering; *Thx*: ProHit Threading Index; *%ID*: percentage of sequence identity between query and template; *chemokine template:* name of the best chemokine template structure; *diS*: number of possible disulfide bonds; *CK Rank*: rank of chemokine fold in the pdb95 control run (only fully covered alignments); *Control*: agreement of model with control structures; *SP*: predicted signal peptide; *SecP*: predicted non-classical secretion; *Loc*: subcellular location of the query predicted by PSORT (Ex, extracellular; Nu, nuclear), *Exon*: number of exons in the gene encoding the query protein; *Iph*: intron phase of the gene encoding the query protein; *ChrLoc*: chromosomal location of the gene encoding the query protein.

The results obtained in the steps explained above (steps 1. to 6. in methods) for proteins B42, N73, G19, L32 are summarized in Table 1 and compared with those obtained for reference chemokine CXCL17. Their corresponding genes were analysed in detail for conservation across different species. In addition, we checked exon organization, chromosomal location and proximity to known chemokine genes, presence of a PolyA sites and Polyadq, transcription factor binding sites of chemokine regulators and gene expression profiles. Their protein sequences were analysed for glycosylation sites and subcellular localization (for details see section 7 in Materials and Methods). The analysis of protein secretion and localization, number of exons, intron phase and chromosomal location for these proteins is also summarized in Table 1. Similar to CXCL17, the B42 and N73 proteins are predicted to be secreted. In contrast, the obtained subcellular localization predictions for G19 and L32 were contradictive. Chemokine genes typically consist of 3–4 exons [28], which coincides with what we observe for the B42 and N73 genes. The results obtained for the discovery of B42 and N73 as putative new human chemokines and their further characterization are described in detail in the following sections.

### Characterization of Chemokine-like Protein B42

B42 (UniProt: Q1T7F1) is 81 amino acids long and contains 6 cysteine residues. By using standard sequence-based methods (BLAST and InterPro; see Materials and Methods for details), we found no significant sequence homology of B42 to any previously characterized protein. B42 was submitted to UniProt/TrEMBL together with four zinc finger proteins (Q76KX8, Q1T7F5, Q76KX9, Q1T7F6). The B42 gene was first automatically labeled as zinc finger protein ZNF528 in Ensembl (release 54) because its protein coding region was located within the untranslated region (intron) of the Kruppel-like zinc finger gene ZNF528. The translated B42 protein does not share sequence identity to any characterized zinc finger protein, however, many zinc finger genes are located in close proximity to the B42 gene on the chromosome. Since Ensembl release 55, it was renamed to ZNF578, which, according to the Ensembl annotation, does not code for a protein, as it is labeled as ‘processed transcript’. The gene annotation of the human B42 gene produced by the automatic Ensembl *genebuild* prediction method did not detect the B42 gene in more recent Ensembl releases (62 and higher) for reasons not specified on their website, although the genomic sequence is identical to the sequence of previous versions. Interestingly, the orthologous chimpanzee and orangutan genes of B42 are still annotated as novel protein coding genes in the current Ensembl release 64.

#### Fitting of the B42 sequence into the 3D chemokine-like fold profile

Table 2 shows the best five hits resulting of threading the B42 protein sequence against the *Chemokine fold library,* which includes all currently available structural information about the chemokine fold. The top two ranking alignments are found with Kaposi’s sarcoma-associated herpesvirus encoded vMIP-I and vMIP-II, which share 24.2% and 26.2% sequence identity with B42, respectively. vMIP-I and vMIP-II are viral chemokines known to block HIV entry through the chemokine receptors CCR3 and CCR5 [29], [30] and, in addition, vMIP-II is able to block CXCR4. Likewise, the chemokine CCL3, found as best scoring human chemokine (rank 3), is reported to block HIV infection through the CCR5 receptor [31] as well as the truncated version of CCL14 [9–74] (rank 4) and the human chemokine CCL5 (rank 5) [32]. Interestingly, all high scoring templates for B42 share the ability of binding to the chemokine receptor CCR5. Besides the anti-HIV function, various different functions have been reported for these template proteins, such as promotion of angiogenesis [29] for vMIP-I and vMIP-II. For CCL5, induction or inhibition of chemotaxis, proliferation [17] and induction of apoptosis at high concentrations [18] have been reported.

**Table 2:** Fold recognition results for the top five hits obtained for B42 with the *Chemokine fold library*.

Rank	Thx	% ID	CK Template	PDB	Chain	pl	fl	UniProt	Ragonist	Rantagonist
1	45.6	24.2	vMIP-I	1ZXT	A	62	69	Q98158_HHV8	CCR8, part. CCR	part CCR5
2	45.1	26.2	vMIP-II	1CM9	B	65	67	VMI2_HHV8P	CCR3	CCR5,1,2, CXC4,XCR1, CX3CR1
3	38.5	21.0	MIP-1A	3FPU	B	62	66	CCL3_HUMAN	CCR1, CCR5	–
4	35.6	18.0	CCL14	2Q8R	H	61	63	CCL14_HUMAN	CCR1,3, CCR4	–
5	30.6	20.6	RANTES	2VXW	A	63	69	CCL5_HUMAN	CCR1,3, CCR5	–

*Rank*: rank of hit within all chemokine structures in the fold library; *Thx*: threading index; *%ID*: percentage of sequence identity between query and template; *CK Template*: PDB template; *PDB*: template identifier in the Protein Data Bank; *chain:* template chain; *pl*: alignment path length; *fl*: template fold length; *UniProt*: UniProt identifier; *Ragonist*: Chemokine receptors for which the template protein is an agonist; *Rantagonist*: Chemokine receptors for which the template protein is an antagonist.

#### Fitting of the B42 sequence into the chemokine-like fold across fold space

Noteworthy, when B42 is threaded against the *pdb95 fold library*, which contains representatives of all protein folds currently available in the PDB, the IL8-like chemokine fold appears as the top number one hit with the template v-MIP-I (Table 3). With the alignment of the B42 sequence into the v-MIP-I template structure, we could observe the possibility of formation of three disulfide bonds in B42. Besides, the other top four hits were considered false positives based on our filtering criteria based on secondary structure and gap content, and fold coverage (see Materials and Methods section 4 for details). These results were strongly indicative of the protein B42 being a possible remote structural homolog of the IL8-like chemokine fold.

**Table 3:** Fold recognition results for the five top fold hits obtained for B42 with the *pdb95 fold library*.

Rank	Conf	sse	gap	fcov	Thx	%ID	SCOP	%Cov	Template	PDB	Chain	pl	fl	UniProt	Pfam
1	HC	+	+	+	45.6	24.2	d.9.1.1	98	vMIP-I	1ZXT	A	62	69	Q98158_HHV8	IL8
2	FP	+	−	+	39.4	21.4	g.3.7.2	100	ergtoxin	1PX9	A	42	42	KGX11_CENNO	Toxin_17
3	FP	+	+	+	34.2	21.9	a.60.1.2	100	Diacylglycerol kinase	3BQ7	D	73	68	DGKD_HUMAN	SAM_2
4	FP	+	−	−	32.7	16.1	c.2.1.1	72	L-Thr 3-dehydrogenase	2D8A	A	81	348	TDH_PYRHO	ADH_N
5	FP	+	+	+	31.7	15.2	a.77.1.2	99	TNF-Receptor 1	1ICH	A	79	87	TNR1A_HUMAN	Death

Only best template per SCOP fold is shown. *Rank*: rank of hit within all structures in the fold library *Conf:* confidence level of prediction (HC, high confidence; FP, false positive); *sse*: all secondary structure elements present in alignment; *gap*: gaps shorter than 10 amino acids; *fcov*: fold coverage based on ratio of fold length/path length (fl/pl); *Thx*: threading index; *%ID*: percentage of sequence identity between query and template; *SCOP*: SCOP family identifier; *%Cov*: percentage of domain coverage; *Template*: PDB template; *PDB*: template identifier in the Protein Data Bank, *Chain*: template chain; *pl*: alignment path length; *fl*: template fold length; *UniProt*: template UniProt identifier; *Pfam*: Pfam description.

#### Modeling B42 into a functional IL8-like chemokine structure

**Figure 2:**
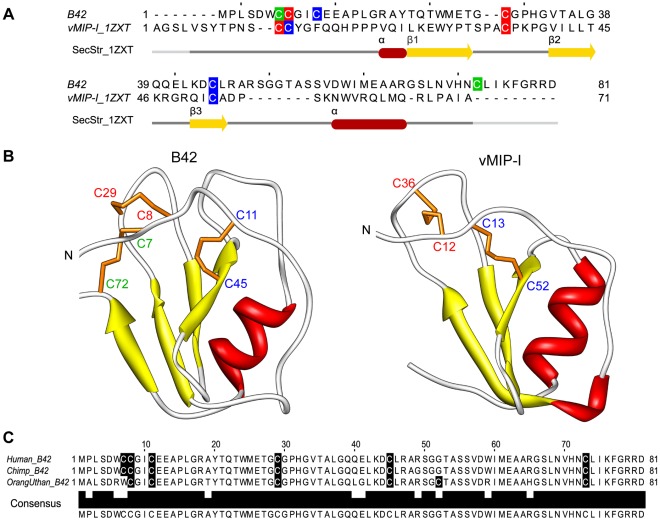
Modelling of the B42 protein as an IL8-like chemokine. A) Sequence-to-structure alignment of B42 with its best scoring template vMIP-I (PDBId: 1ZXT). The secondary structure of vMIP-I is indicated below (yellow arrows for β-strands, red cylinders for α-helices, and dark grey line for coil regions; the light grey line indicates no atom coordinates for those residues in template). Cysteines are highlighted in boxes coloured (green, red, and blue) according to their pairing in each protein sequence. B) The 3D model of B42 (left) is compared with the X-ray structure of vMIP-I (right) used as template. Disulfide bonds are shown as orange sticks, and corresponding cysteines are labelled according to their pairing with the same colour code as in panel A. C) Sequence alignment of human B42 with its orthologous proteins (Ensembl release 54). Cysteines are highlighted in black boxes. The sequence identity between human and chimpanzee (Chimp_B42: ENSPTRG00000034188) is 98%, and 91% with oranguthan (OranUthan_B42: ENSPPYG00000010339).

The top threading hit vMIP-I (PDBId: 1ZXT, resolution: 1.7 Å; Table 3) was used as template to model B42 as an IL8-like chemokine structure (Figure 2 A, B). Although B42 and vMIP-I share low sequence identity (24.2%), once B42 is modelled into the chemokine-like scaffold, it can be seen that structurally relevant amino acids forming the core of the fold are well conserved in 3D. B42 presents a small insertion of 7 residues between the third beta strand (β3) and the C-terminal alpha helix with respect to v-MIP-I, and it has a longer C-terminus, which includes a cysteine residue. The six cysteines contained in B42 are coming together in 3D to form disulfides, which interestingly are in non-canonical sequence and structure positions seen in known chemokines. The three disulfide bonds predicted in B42 can be seen in Figure 2B (cysteines C7 and C72, C8 and C29, C11 and C45), which shows the vMIP-I template structure in comparison with the 3D model of B42. Interestingly, our posterior mass spectrometry experiments with the B42 protein showed a monoisotopic mass of 8,931.2085 Da, which is indicative of a yield of 3 intra-molecular disulfide bonds in the B42 protein (see “Experimental characterization of B42” below). These experimental results further support our model and hypothesis that B42 may fold into a functional IL8-like chemokine architecture.

The 3D model of B42 was energy refined in a 10 ns molecular dynamics (MD) simulation and analysed by using the 3D models of vMIP-I and CXCL17 as comparative references (Materials and methods section 6). The CXCL17 chemokine has low sequence identity to other known chemokines, and it was characterized by threading and confirmed experimentally to have an IL8-like chemokine fold [12]; therefore it constitutes a good reference for comparison. In general, the B42 and vMIP-I models showed similar behaviour during the MD trajectory (Figure S1 A). Although B42 has a 7 amino acid small insertion and a flexible tail of 8 amino acids at the C-terminus, which are not present in the reference model, both, the coil and backbone RMSD curves of B42 are in the same range as in the reference. Similar to the reference model, the RMSD values of B42 converge over time, indicating that the overall fold stays compact along the simulation. These results are also comparable to the results obtained for our reference model CXCL17 (Figure S1 C). These RMSD values (0.2–0.3 nm) were assumed as an upper boundary for the secondary structure RMSD values observed in the putative chemokine models. These results further support the hypothesis that the B42 sequence may adopt a compact IL8-like chemokine fold.

In order to quantify the residue energetic contributions in the B42 chemokine-like model, we calculated contact energies per residue and compared them to those of the vMIP-I model. The contact energy decomposition plot for B42 compared to vMIP-I shows a general agreement in the energetic contributions of the corresponding residues. In particular, it can be observed that the secondary structure regions and the core of the protein present a good agreement of the contact energies, indicating that the packing of the core of B42 is comparable to the packing of the vMIP-I template (Figure S2 A). We calculated the coefficient of determination (R^2^) from the contact energy pairs of the B42 model and the reference model of vMIP-I, and we obtained an R^2^ value of 0.63 (Figure S3 A). We also did this analysis for the vMIP-I using as reference a mutant of CXCL8 (PDBId: 1ICW), and we obtained an R^2^ value of 0.43 (Figure S3 C). We selected this CXCL8 mutant as reference, because it has one disulfide bond at a non-canonical position when compared to the rest of the chemokine protein family, and it has low sequence identity (19%) to vMIP-I. The R^2^ value obtained for B42 is higher, which is indicative of the good packing of the core in the B42 model compared to known chemokines and gives high confidence to its predicted structural resemblance towards the IL8-like chemokine fold.

The possibility that B42 could be a lost remote homolog of the chemokine protein family was further explored by searching for the presence of chemokine-like features in its protein and gene sequence.

#### B42 protein sequence and gene analysis

**Figure 3:**
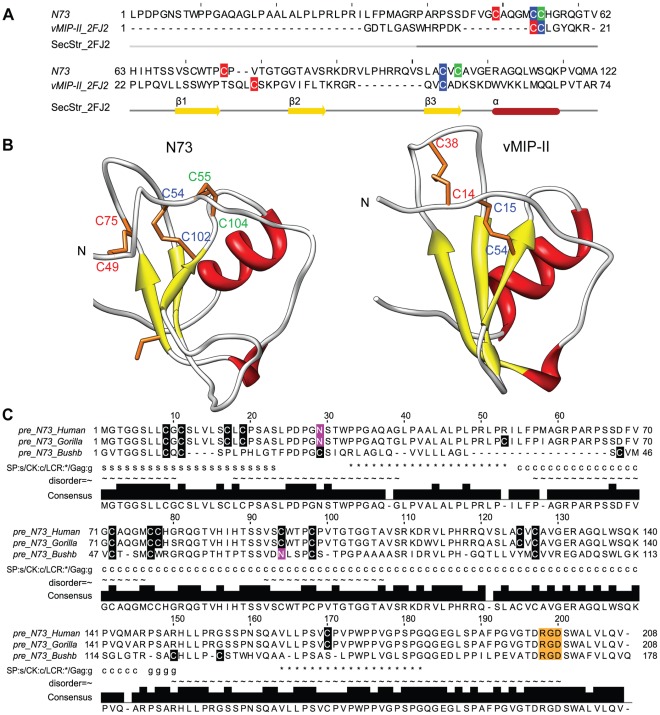
Modelling of the N73 protein as an IL8-like chemokine. A) Sequence-to-structure alignment of the mature N73 (without signal peptide) with its best scoring template vMIP-II (PDBId: 2FJ2). The secondary structure of vMIP-II is indicated below (yellow arrows for β-strands, red cylinders for α-helices, and dark grey line for coil regions; the light grey line indicates no atom coordinates for those residues in template). Cysteines are highlighted in boxes coloured (green, red, and blue) according to their pairing in each protein sequence. B) The 3D model of N73 (left) is compared with the X-ray structure of vMIP-II (right) used as template. Disulfide bonds are shown as orange sticks, and corresponding cysteines are labelled according to their pairing with the same colour code as in panel A. C) Sequence alignment of the human N73 precursor with its orthologous proteins. Cysteines are highlighted in black boxes. Predicted N-glycosylation sites are shown in pink boxes, and the RGD motif in orange boxes. Under the alignment, ‘s’ indicates location of signal peptide (SP), ‘*’ indicates location of low complexity region (LCR), ‘c’ indicates location of predicted chemokine domain, ‘g’ indicates motif for glycosaminoglycan attachment at a serine residue, and ‘∼’ indicates location of disordered region predicted by GlobPlot. The sequence identity between human and gorilla (pre_N73_Gorilla: ENSGGOP00000010336) is 96%, and 45% with bushbaby (pre_N73_Bushb: ENSOGAP00000012479).

In the B42 protein sequence, we did not identify any signal peptide or transmembrane region (see Materials and Methods section 2). At present, one chemokine without signal peptide is known to be transported to the nucleus [33] and so far no chemokine lacking the signal peptide has been reported to be secreted. However, there are some cytokines like IL-1α, IL-1β and IL18 that are leaderless secreted by a non-classical secretion pathway [34]. For this reason, we analysed possible leaderless secretion of the B42 protein with SecretomeP [35]. We obtained a high score, 0.8, which indicates that leaderless secretion of B42 might be possible (*i.e.* minimum value of 0.5). Also WoLF PSORT [36] predicts extracellular location for B42 with a very high confidence (31 out of the maximal possible 32 nearest neighbours are extracellular localized). We also analysed the B42 sequence with the ELM server for possible glycosaminoglycans (GAG) interaction sites, as it is known that GAG interaction is a common feature of chemokines [37]. We identified one putative GAG attachment site, RSGG, in residues 49–52. Our search for B42 orthologous with the Ensembl’s automatic annotation pipeline (Release 54) [38] identified one in chimpanzee and another one in oranguthan (98% and 91% protein sequence identity to B42, respectively), which are also still functionally uncharacterized (Figure 3C).

The B42 gene lies within the same chromosomal region (19q13.41) as two other known chemokines (CXCL17 and CCL25), which are found at 19q13.2. Similar to many known chemokines, the B42 coding transcript originates from 3–4 exons. Another common feature of chemokines is that the exon boundaries have similar intron phases (position of the codon within the intron). They have the intron phase 1 between exon 1 and 2, and phase 2 between exon 2 and 3 [39]. Similarly, the protein coding region of B42 starts at the end of exon 2 and ends with exon 3 presenting a phase 2 intron boundary between exon 2 and 3 (Table 1 and Figure S4 A). Furthermore, the promoter region of B42 was analysed for transcription factor binding sites known from other chemokines, and we found binding sites for NF-IL6, NF-kappaB, AP1 (C-Jun) and INF-1 [40]–[46]. In addition, we found the CK1 motif that was previously described as a chemokine specific motif found in the chemokines MIP-1α and MIP-1β (Table S3) [43].

In summary, the results obtained from our computational analysis strongly support that B42 may adopt an IL8-like chemokine fold as preferred from all known folds currently represented in the PDB. Folded into a chemokine architecture, B42 shows good packing and energetics comparable to other experimentally known chemokine structures. In addition, in this predicted 3D scaffold, B42 is able to form three disulfide bonds, which are structural features known to be necessary for chemokines to fold and function. The fact that these three disulfide bonds are not conserved in 3D with respect to other known structures of members of the chemokine family, and also the low sequence similarity of B42 to known family members could explain that B42 has escaped annotation by sequence-based methods. Furthermore, the identification of functional chemokine-like features in the B42 protein and gene sequence such as GAG interaction site, number of exons, intron phase, chromosomal proximity to known chemokines and similar transcription factor binding sites, all together add confidence to our structural prediction of B42 being a possible remote homolog of the IL8-like chemokine family. In terms of its putative function as a chemokine, a remarkable observation is that B42 is a primate specific protein, and that all the five best ‘high confidence’ template structures obtained in our threading experiments (vMIP-I, vMIP-II CCL3, CCL14, CCL5) are reported to have anti-HIV functions. All in all, our findings make us hypothesize that B42 could be a putative chemokine that could inhibit HIV infection, which constitutes a very interesting testable hypothesis.

### Characterization of Chemokine-like Protein N73

N73 (UniProt: Q71RG6) is 208 amino acids long and contains 12 cysteine residues. Sequence analysis using standard sequence-based methods (see Materials and Methods) did not identify any statistically significant sequence homology of N73 to any previously characterized protein. N73 was first identified as a gene related to cancer development and progression in a large-scale cDNA transfection screening. The transfection with N73 cDNA resulted in inhibition of growth in the Hepatoma 7721 cell line and stimulation of growth in the fibroblast cell line NIH3T3 [47].

The same computational analysis performed for B42 explained above was also carried out for the N73 protein (for details see Supporting Information Text S1 section II). The fitting obtained for the N73 sequence into the chemokine fold using both the *Chemokine fold library* and the *pdb95 fold library* was, like in B42, strongly indicative of the protein N73 being a possible remote structural homolog of the IL8-like chemokine family (Table S4 and S5). The top threading hit vMIP-II (PDBId: 2FJ2; 2.3 Å and 23.2% sequence identity) was used as structural template to model N73 as an IL8-like chemokine. Compatible with the proposed chemokine fold, in this structural framework there are two possibilities of disulfide bond formation (Figure 3 A and B). The MD optimization of our N73 chemokine-like model, its comparison with reference models of vMIP-II and CXCL1, and the contact energy analysis performed were indicative of a good packing of the core in the predicted 3D scaffold (Supporting Information Text S1 section II, Figure S2 B, Figure S3 B).

The analysis of the N73 sequence uncovered several chemokine-like properties such as the presence of a signal peptide for secretion, a GAG attachment site as well as an N-linked glycosylation site (Figure 3C; see Supporting Information Text S1 II for details), which are also known from other chemokines like CCL2 [48]. We also found transcription factor binding sites known from other chemokines in its promoter region (Supporting Information Text S1 II and Table S6). Interestingly, N73 was previously identified to be involved in cancer development and progression, which could be caused by the RGD-integrin binding motif in its sequence (Figure 3C). By integrin interaction with this motif, N73 might acquire anti-angiogenic function. On the other hand, the analysis of orthologous showed that, similar to B42, N73 is a primate specific protein (Figure 3C). The N73 gene (Gene ID: ENSG00000184334 in Ensembl release 61) comprises 3 exons like many other chemokines; however, the protein coding region lies only on the first one. The open reading frame is located on chromosome 8q24.3. At present, no other chemokines are known to be located on this chromosome. Close to the N73 gene, an orphan G protein coupled receptor (GPR20) is found (Figure S4 B). The annotation of the human N73 gene produced by the automatic Ensembl *genebuild* prediction method did not detect the N73 gene in more recent Ensembl releases (62 and higher) for reasons not specified on their website, although the genomic sequence is identical to the sequence of previous versions. Interestingly, the orthologous gorilla and bushbaby genes of N73 are still annotated as novel protein coding genes in the current Ensembl release 64.

In summary, the results obtained in our computational analysis support our prediction that N73 may adopt a functional IL8-like chemokine fold. Furthermore, the fact that N73 resembles the structures of the chemokines vMIP-II and vMIP-I that are known to have anti-HIV functions suggests that also N73 might share these properties, which constitutes a very interesting testable hypothesis.

### Experimental Characterization of B42

In order to corroborate our predictions, we carried out gene expression analysis, protein expression studies, and protein structure investigations by means of Circular Dichroism (CD), Fourier transform infrared spectroscopy (FTIR) and Mass Spectrometry (MS).

#### Gene expression analysis

**Figure 4:**
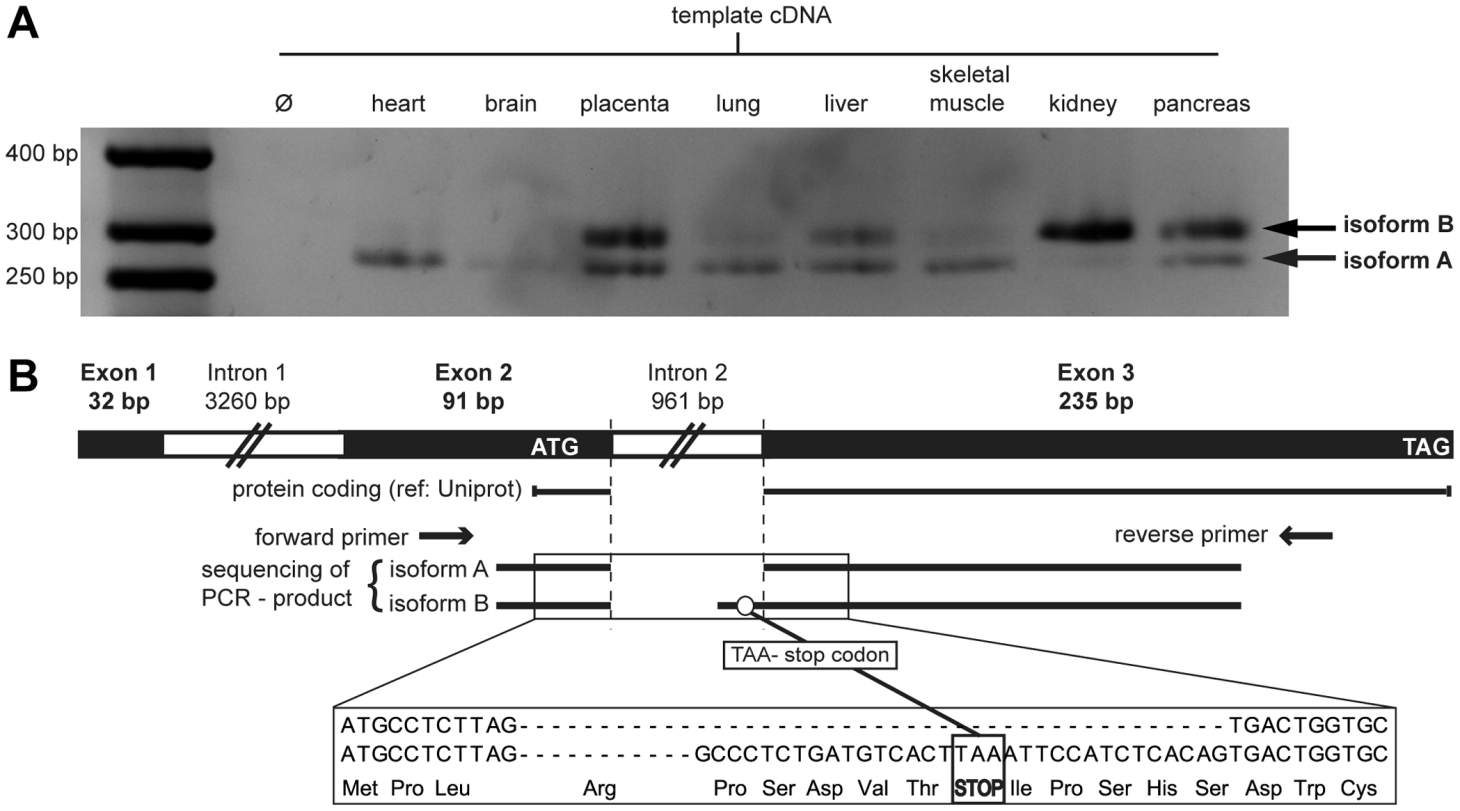
Expression and gene structure of the B42 isoforms. A) Expression of two B42 isoforms in human tissues (expected size for isoform A: 272 bp). B) Sequencing results of the two different B42 isoforms. Isoform A codes for the full length B42 protein, whereas Isoform B contains a stop codon after 9 residues.

Gene expression studies utilizing cDNA isolated from different tissues and subsequent sequencing of the PCR products identified two B42 isoforms. Only isoform A is protein coding and expressed in placenta, heart, lung, liver, pancreas, skeletal muscle and weakly in brain (Figure 4A). Isoform B exhibits a stop codon within the additional longer exon 3 sequence encoding a short peptide of 9 residues, which is expressed mainly in kidney, placenta, pancreas, liver and weakly in lung and muscle (Figure 4B). Bone marrow cDNA was also tested but no B42 expression was found (data not shown).

#### In vivo protein expression and localization

**Figure 5:**
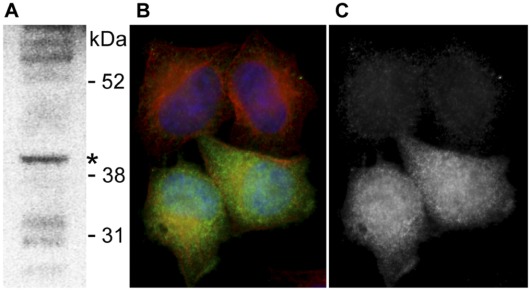
Protein localization results of B42. A) Westernblot analysis of the C-terminal GFP-tagged B42 transgenic cell pool using an antibody to GFP. A weak band of the expected size 39.8 kDa (GFP-tag: 31 kDa+B42 8.8 kDa) was detected (marked with *). B) Immunoflourescent staining of the transgenic cell line. Two positive and two negative cells of the cell pool are shown. Localization of the C-terminal GFP-tagged B42 protein is mainly in the cytoplasm of HeLa cells: GFP - green, alpha-tubulin - red, DNA (DAPI) – blue. C) Only GFP-signal of B is shown in grey scale.

A transgenic HeLa cell line was generated carrying the LAP-tagged B42 gene in a bacterial artificial chromosome (see Materials and Methods section 8 for details). The expression of the transgene was confirmed by Westernblot (Figure 5A) showing that B42 is expressed and translated into protein. Our results confirm the existence of the translated protein B42 and thus prove wrong its previous Ensembl labeling as a ‘processed transcript’ (rel. 55–61) and the complete removal of the B42 gene in more recent Ensembl releases (62 and higher) (see Figure S4 A).

Immunoflourescent staining and imaging of the transgenic cell line showed positive cells at a low percentage (2%). The localization of B42 is ubiquitous, mainly cytoplasmic (Figure 5B, C) and does not change over the different cell cycle phases (data not shown). No indication for secretion of B42 in the transgenic cell line was observed. However, a missing additional stimulus or hindrance by the GFP-tag could explain that B42 does not seem to be secreted in our analysis. Although not observed in our experiments, currently available predictors support the hypothesis that B42 could be leaderless secreted. It could also be possible that B42 may act like PESKY, a non-secreted mouse isoform of the chemokine CCL27, which is able to modulate transcription in the nucleus [33].

#### Analysis of disulfide bond formation by Mass Spectrometry

**Figure 6:**
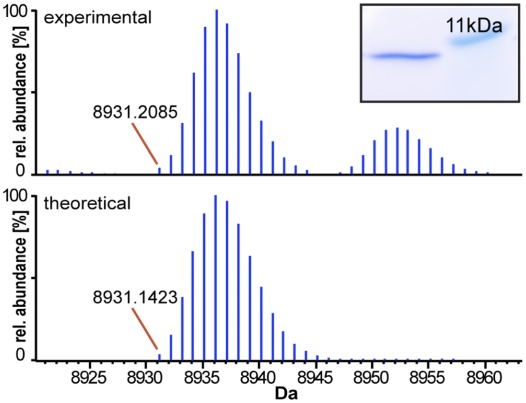
Deconvoluted mass spectrum of B42. Upper panel: Deconvolution of experimental spectrum with monoisotopic peak labelled. Inlay: SDS-page gel electrophoresis results to check B42 protein recovery. Lower panel: Theoretical isotopic distribution for [GA-B42] assuming 3 disulfide bonds, with monoisotopic peak labelled.

The *in vitro* expressed B42 protein (glycine-adenine-B42) was analyzed by SDS-page gel electrophoresis to verify B42 protein recovery (Figure 6). The same construct was analyzed by Mass Spectrometry. The result of the experimental deconvoluted mass spectrum was then compared to the theoretical isotopic distribution for glycine-adenine-B42 assuming 3 disulfide bonds based on our 3D models. The experimental value (8,931.2085 Da) deviates by only Δm = +0.0662 Da from the expected theoretical value (8,931.1423 Da). Accordingly, the experimental monoisotropic mass of B42 indicates that all 6 cysteines are oxidized and thus involved in the formation of three disulfide bonds as predicted.

#### Analysis of secondary structure content by CD and FTIR

**Figure 7:**
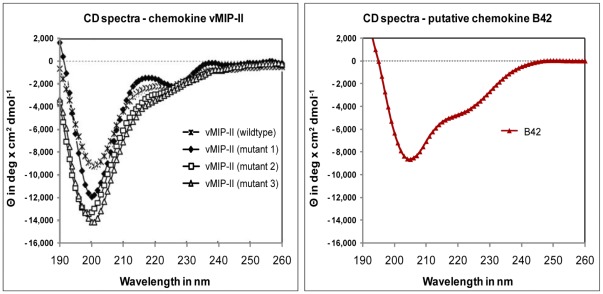
Circular dichroism (CD) spectra of vMIP-II and B42. The CD spectrum of chemokine vMIP-II taken from literature [68] is shown (left) in comparison to the spectrum measured for B42 (right).

The fractions of secondary structure elements calculated for B42 from the Circular Dichroism (CD) and Fourier transform infrared (FTIR) spectroscopy spectra are in agreement with the theoretical fractions calculated for our chemokine-like 3D model (Table 4), which confirms our structural predictions. The comparison of the CD spectra of B42 with the spectra of the chemokine vMIP-II shows a similar pattern suggesting similar secondary structure content in both proteins (Figure 7). The small difference observed in the content of beta sheets might be due to: i) an underestimation of the secondary structure content of our model (i.e. extended regions not defined as beta strands), and ii) the possibility of oligomer formation, which we do not consider when calculating the percentages of secondary structure elements for our chemokine-like 3D model of B42, as B42 is modelled as monomer. Oligomerisation is a common feature of chemokines. In particular, vMIP-I and vMIP-II are known to dimerize by forming inter-chain beta sheets between their N-termini [49] and the same dimerization mode was also observed for the chemokines CCL3, CCL5 and CCL14 [49] found as best ranking human templates for B42 (Table 2).

**Table 4:** Predicted and experimentally determined secondary structure content of B42.

	% Predicted from model	% Measured by CD	% Measured by FTIR
**α helical**	10	15	14
**β strand**	17	27	45
**Turn/β turn**	20	19	21
**Unordered**	53	35	19

Secondary structure fractions (in percentage and number of amino acids) calculated for the 3D model of B42 and compared with the experimentally determined fractions obtained by Circular dichroism (CD) and Fourier transform infrared (FTIR) spectroscopy.

In summary, we have shown that the B42 gene is expressed in several human tissues, and encodes an *in vivo* translated folded protein. This contrasts with the fact that the B42 gene product was previously labelled as a ‘processed transcript’ in Ensembl and as ‘retired’ in more recent releases. The experimentally measured secondary structure content of the B42 protein is in agreement with that calculated for our chemokine-like model of B42 and the one reported for vMIP-I and other chemokines. Furthermore, mass spectrometry experiments corroborate the existence of three disulfide bonds, which our model predicts to be determining the folding of the IL8-like chemokine architecture and that could be indicative of a putative chemokine function for B42. All in all, our experimental results further support our prediction of B42 adopting an IL8-like chemokine fold, and they substantiate our structure-based functional hypothesis, which proposes B42 as a putative novel human chemokine.

### Conclusions

We have developed a new computational approach for automatic proteome-wide identification of novel chemokines based on three-dimensional properties of this protein family. In this 3D profile-based methodology, we combine fold recognition methods with automatic scaffold-based disulfide mapping to detect structural and functional patterns in 3D space indicative of a preference for a functional IL8-like chemokine fold. We apply our methodology to several thousands of so far uncharacterized human proteins to identify potential remote homologs of the chemokine protein family that may have not yet been discovered due to their low or inexistent sequence similarity to already characterized family members, and possibly also due to their non-canonical cysteine patterns in sequence and in 3D. We describe the discovery of two new proteins, B42 and N73, which we predict with high confidence to resemble the IL8-like chemokine fold of vMIP-I and vMIP-II, despite their respective low sequence similarities to known members of the chemokine protein family. Based on our computational results and in the obtained experimental supporting evidence, we propose the B42 protein to be a new structural member of the IL8-like chemokine fold family and possibly a new human chemokine. Based on the observation that B42 and N73 are primate specific proteins and because of their structural resemblances towards known anti-HIV chemokines, we postulate the possibility that both proteins might have an HIV inhibitory function. Furthermore, based on the sequence features observed for N73, we hypothesize that N73 might be able to promote tumor necrosis in cancer, like known angiostatic chemokines. Further experimental analyses will be necessary to support these hypotheses.

Our findings are relevant for the signature of the chemokine family, as it gets enriched with the discovery of each new family member, and it may then help to identify new members. Each of these discoveries may shed light on the molecular mechanisms of the functions of the chemokine protein family, an understanding essential for the development of treatments for pathological processes where these proteins are involved. Besides, the 3D family profile-based methodology presented here and the results obtained denote the significance of structure-based methods as complementary approaches to current sequence-based approaches for genome-wide detection of similarities among proteins at very low or even non-existent levels. The 3D scaffold-based profile concept applied here could be easily extended to other disulfide-containing protein folds and, furthermore, to any other fold family for which structure-to-function markers may be described and automatically screened in 3D. More than 500 of the currently known protein folds contain disulfide bonds, and some are associated to inflammatory responses, angiogenesis or cancer processes, which certainly represent very interesting targets for future discovery. Thus, the concept of our methodology has important implications for helping in the discovery of remote homologs of protein families with interesting properties for medicine and technology developments. All in all, our approach may be of help in functional genomics efforts and support protein annotation attempts for proteins that fall into the twilight-zone of the protein sequence-to-structure universe.

## Materials and Methods

### 1) Sequence Data Extraction

The sequence dataset used in our study was extracted from the UniProt Knowledgebase (Release 14.9) consisting of UniProtKB/Swiss-Prot Release 56.9 and UniProtKB/TrEMBL Release 39.9. All human protein sequences labelled as *“unknown”*, *“orf”, “hypothetical”, “uncharacterized”* or *“putative”* that contained at least two cysteine residues in their sequence were selected for our studies.

### 2) Automated Pre-filtering

#### Structural annotation check

BLAST against the PDB was used to detect query sequences or parts of them that could be already structurally characterized. Alignments longer than 50 residues with an e-value ≤0.0005 and with sequence similarity >30% were considered annotated and were, therefore, discarded for further analysis.

#### Signal peptides

Signal peptides (SP) were predicted in the query sequences using the consensus of two different prediction methods: PrediSi [50] and SigPfam [51]. We tested both methods using default parameters and a dataset of 24 protein sequences. Based on the annotated positions of the signal peptides in the test dataset, we used the scores calculated by each method to assign confidence groups to combine the results of both methods and thus remove those regions predicted as signal peptides with high confidence (for details see “Consensus signal peptide prediction” in Supporting Materials and Methods).

#### Transmembrane regions

We predicted transmembrane (TM) regions in the query sequences using the consensus of two different prediction methods: TMHMM [52] and Memsat [53]. As the average length of a TM helix is known to be 21 residues, and 94% of all TM segments have a length between 17 and 25 residues [54], we assigned confidence groups for the consensus prediction based on the predicted length of the TM region. According to the TM segment length, we assigned confidence groups for each predicted membrane segment. For high and medium confidence predictions, the predicted TM region was removed from the query sequence (for details see “Consensus transmembrane prediction” in Supporting Materials and Methods).

#### Cysteine content and sequence length

The cysteine content of the remaining sequences was evaluated, and only those sequences with two or more cysteine residues and a minimum sequence length of 55 amino acids were selected for further analysis.

### 3) Proteome-wide 3D Profile-based Chemokine Search

#### Chemokine 3D fold descriptor

As three-dimensional descriptor of the IL8-like chemokine architecture, a fold library was built (*Chemokine fold library*) that contained as templates all structures available from the Brookhaven Protein Data Bank (PDB) that are classified as IL8-like chemokine fold in SCOP (SCOP: d.9). This library consisted of 270 structural templates of chemokines.

For the pre-filtered query sequences, sequence-to-structure alignments and 3D models were obtained using the fold recognition algorithm ProHit (ProCeryon Biosciences, ProHit Professional V 2.2.2) [25], [26]. ProHit was used with parameters as previously described for gap handling, scoring, and evaluation of the alignments [12]. The final overall threading ranking was calculated based on the ProHit Threading index (Thx), which is a combination of sequence similarity, residue-residue (z_pair) and residue-solvent (z_surf) interaction z-scores normalized by the query sequence length. A cutoff of 25.3 for the threading index was considered as representative cutoff for the chemokine family, as it was obtained by threading the remote member of the chemokine family CXCL17 [12] (Table S7). The percentage of amino acid sequence identity (%ID) between query and template sequences as well as the percentage of SCOP domain coverage (%Cov) of the query sequence onto the template structure were calculated. To exclude false positives (FP), we used the ratio between the template fold length (fl) and the number of aligned residues in the sequence-structure alignment (path length: pl) as previously described [12] to reject those sequence-structure alignments not covering the full length of a given template fold were rejected. Hits with values 0.6≤fl/pl≤1.3 were considered to be of high confidence (HC), and all other were considered to be false positives (FP). In addition, high confidence hits should have all secondary structure elements of the template covered by the query sequence, and the sequence-to-structure alignment should not contain gaps longer than 10 amino acids. Alignments not fulfilling these criteria were rejected.

#### Automatic scaffold-based disulfide mapping

All possible cysteine pairs in the query proteins were automatically checked for disulfide bond formation possibilities based on the obtained query-sequence to template-structure alignment for each HC threading hit. The template structures were automatically mapped for possibilities of disulfide bond formation in positions where the query sequence had cysteine residues using the following criteria:

i) d_(Cα1, Cα2)_≤10 Å and d_(Cβ1, Cβ2)_≤9 Å: The distance between the Cα atoms of two cysteines should be ≤10 Å and between their Cβ atoms ≤9 Å.
ii) d_(Cβ1, Cβ2)_−d_(Cα1, Cα2)_≤1 Å: The distance between the Cβ atoms should not be much bigger than the distance between the Cα atoms (*i.e.* the side chains should not point in opposite directions from each other; which would not enable disulfide bond formation).
iii) Two cysteines should be 3 amino acids apart in the protein sequence to be able to form a disulfide bond.

A disulfide bond in the query protein was considered to be possible when all these criteria were fulfilled. In cases where cysteines of the query sequence were aligned to a gap or an unresolved residue in the template, the coordinates of the closest left and right sequence neighbour in the template were used to measure possible pairwise cysteine distances.

### 4) 3D Profile Search Across Fold Space

For the control threading experiments, a fold library containing all structures in the PDB filtered at 95% sequence identity was used in order to avoid redundancy and to account for all representative 3D architectures (*pdb95 fold library*). This library consisted of 23,833 structures. The threading calculations and ranking were performed as described above for the chemokine-like 3D fold descriptor. The following selection criteria were applied to decide on high confidence chemokine-like candidates:

#### Fold and SCOP domain coverage

As described above for the chemokine-like 3D fold descriptor, only sequence-to-structure alignments corresponding to fully covered template folds were considered. For this, and as previously reported [12], hits with values 0.6≤fl/pl≤1.3 were considered to be of high confidence, and all other were considered to be false positives (see section 3 above for details). The same criteria as explained above were used for allowing gaps in the query-to-template alignment and discard false positive hits.

The structural templates in the *pdb95 fold library* may contain more than one SCOP domain in the same PDB chain; we therefore used an additional criterion to discard those SCOP domains in the results list that were not fully covered: the SCOP domain coverage (%Cov). All folds with an SCOP domain coverage lower than 70% were considered incomplete hits and discarded. The top 20 SCOP domains were considered, and only those query sequences that had the chemokine fold ranked as first or second high confidence hit after applying the fold and SCOP domain coverage filter were taken for further analysis.

### 5) InterPro Analysis

The InterPro database (http://www.ebi.ac.uk/interpro/) was used to look for signatures in the selected sequences that could contradict our predictions. InterPro integrates predictive models or signatures representing protein domains, families and functional sites from diverse source databases such as Gene3D, PANTHER, Pfam, PIRSF, PRINTS, ProDom, PROSITE, SMART, SUPERFAMILY and TIGRFAMs [27].

### 6) 3D Model Building and Analysis

#### Comparative modelling and refinement

The MODELLER package [55] was used to generate 3D models of the query proteins based on the sequence-to-structure alignments obtained from threading calculations with the *pdb95 fold library* (Table S2). The MOE2008.10 program (Chemical Computing Group, Quebec, Canada) was used to connect unbound cysteines where disulfide bond formation was predicted and to adjust side chain rotamers when needed. The resulting models were energy minimized the with the Amber99 force field [56] in MOE. Good models were selected based on two criteria: proper formation of secondary structure regions with respect to the template structures and good disposition of inserted regions. Models were refined by applying 10 ns molecular dynamics (MD) simulations, which were performed using GROMACS 3.3 [57], [58] with the G53a6 force field (GROMOS96.1) [59] using a previously described protocol [60]. The refined models were graphically analysed using VMD [61], MOE2008.10 and Discovery Studio 1.7.

#### Reference models

The 3D models of the template proteins vMIP-I and vMIP-II were built by using the backbone of their experimental structures (PDBId: 1ZXT and 2FJ2, respectively) and adding the corresponding side chains with MODELLER [55]. The model of CXCL17 was generated using MODELLER and the X-ray structure of CCL17 as template, as it is the best compatible chemokine template structure currently available in PDB (PDBId: 1NR4; 1.72 Å and 21.4% sequence identity) (see Table S7). The obtained models were refined using the protocol described above.

#### Analysis of models

The refined models of the putative chemokines were evaluated by: 1) Analysis of their RMSD plot through the MD simulation, 2) comparison of the RMSD plots of the simulation of the putative chemokine models with the RMSD plot of the corresponding reference models (vMIP-I and vMIP-II, respectively, as well as CXCL17), and 3) graphical analysis of the overall structure after the simulation and 4) comparison of the packing of the protein core by analysis of the energetic contributions of each residue in the templates with the corresponding residue in the putative chemokine models (contact energy correlation; see below).

#### Contact energy calculation and correlation analysis

The contact energies per residue of coordinate snapshots taken from the MD simulation were calculated using the contact energy function built in MOE2008.10 and as previously reported [60]. To analyse the correlation of the *per residue* contact energies between reference templates and chemokine-like models, we calculated a structure-based sequence alignment for each chemokine model with its respective reference template using the structure alignment algorithm ProSup in ProHit. This alignment was then used to calculate the coefficient of determination (R^2^) for the contact energy pairs of structurally corresponding residues in the alignment (gaps were not considered). To estimate the order of correlation that can be expected from a known chemokine, the same analysis was done for the reference chemokines with the X-ray structure of a CXCL8 mutant (PDBId: 1ICW).

### 7) Detailed Protein and Gene Analysis

Orthologous proteins were identified using the Ensembl automatic annotation pipeline (Release 58) [38] and analysed with the Jalview 2.4 alignment viewer {Waterhouse, 2009 #129}. Exon organization, chromosomal location and proximity to known chemokine genes, presence of a PolyA site (using Ensembl, Polyah.pl (softberry) and Polyadq [62]), transcription factor binding sites of chemokine regulators [40]–[45] and gene expression profiles (GeneNote expression study [63]) were checked. Protein sequences were analyzed for eukaryotic linear motifs and glycosylation sites using the functional site detection server ELM [64] with the ‘extracellular’ filter. Subcellular localization was predicted with WoLF PSORT [36]. In case no signal peptide was present leaderless secretion was checked using SecretomeP [35].

### 8) Experimental Methods

#### Gene expression analysis (PCR)

The mRNA expression of B42 was analyzed by PCR with human cDNA templates from eight different tissues (heart, brain, placenta, lung, liver, skeletal muscle, kidney, pancreas) using Human MTC Panel I (Clontech, Catalogue No. 636742, LOT No. 8082935A) with forward and reverse primers 5′-CCTAAGGAAGAAGCCTAGAAGAGG-3′ and 5′-CAGGCAGTTGTGCACATTAAG-3′, respectively (annealing temperature 58°C).

#### Generation of a BAC transgenic HeLa cell line and tag-based protein localization

Transgenic HeLa cell lines [65] were generated carrying the tagged gene of interest in a BAC (bacterial artificial chromosome) using the methodology described by Poser et al [65].

#### Protein expression and purification

The gene encoding the mature B42 protein was synthesized and cloned in an expression plasmid pETMM-60. The expression and purification was done in *E. coli* Origami B cells (Novagen) (for disulfide bonds formation in cytoplasm because of a glutathione S transferase mutation) as described by Magistrelli [66] using a His-tagged NusA fusion protein as to make the putative chemokine as fusion partner soluble. The putative chemokine was cleaved from NusA at the TEV cleavage site with TEV protease at a 1∶100 concentration overnight at 18°C. After cleavage the chemokine construct keeps a GA-addition of the cleavage site at the N-terminus. The B42 protein was then purified and afterwards concentrated. The expression and purification of the right construct was confirmed by SDS-Page and mass spectrometry.

#### Validation of disulfide bond formation by Mass Spectrometry

Mass spectrometry was used to validate the formation of disulfide bonds. Experiments were done with a protein concentration of 28.29 µM/mL in 1×PBS and at a pH of 7.3. Desalting and concentration was performed with a C-18-µZipTip (Millipore, Billerica, USA) supplemented with Poros Oligo R3 (C-18 reversed phase, Applied Biosystems Germany, Darmstadt, Germany) prepared in-house. The sample was acidified by addition of 2 µl 30% formic acid to 18 µl protein solution, washed three times with 5% methanol 0.1% formic acid and eluted in 40% methanol 0.1% formic acid. Analysis of the sample was carried out with on an Orbitrap XL mass spectrometer (ThermoScientific, Bremen, Germany) by direct infusion with a TriVersa Nanomate robot using 2.1 µm nozzle chips (Advion BioSciences, Ithaca, USA). Data was acquired in profile mode at a resolution of 60000 at 400 Th and automatic gain control was enabled with a target value of 5*10 E5. Spectrum deconvolution was performed with the XtractAll add-on to the Xcalibur software suite (Thermo Scientific, Bremen, Germany).

#### Circular dichroism spectroscopy

For determination of secondary structure fractions, Circular Dichroism measurements were carried out in the far UV-range (190–260 nm) on a Jasco J-815 Circular Dichroism Spectropolarimeter using cuvettes of 0.2 mm path length. The protein was used at a concentration of 28.3 µM/mL at 20°C in 1×PBS, pH 7.3. The results were averaged over ten repetitive scans at a scan rate of 1 nm/s. Ellipticity was normalized to molarity using a mass of 8,931.2085 Da as determined by mass spectrometry. The secondary structure content was calculated using CONTINLL and SELCON3, both provided in the CDpro software package.

#### Fourier transform infrared spectroscopy

Fourier transform infrared (FTIR) spectroscopy was used to obtain the overall fractions and alpha helical and beta sheet secondary structure elements in the measured protein. The amide I band was decomposed into peaks with Peakfit and the percentage of secondary structure elements was calculated using the factors described by de Jongh et al [67].

## Supporting Information

Figure S1 **Results of MD simulations for chemokine-like models of** **B42, N73, and the reference models of vMIP-I, vMIP-II and CXCL17.** A) RMSD along the MD simulation of the chemokine-like B42 model (left) and vMIP-I model (right). B) RMSD along the MD simulation of the chemokine-like N73 model (left), and vMIP-II model (right). C) RMSD along the MD simulation of the CXCL17 model. Black lines correspond to backbone, margenta to secondary structure elements, and blue to coil regions.
(TIF)

Figure S2 **Contact energy decomposition plots.** Contact energies of the residues in the putative chemokine models are compared to the corresponding residues in their template structures (gaps are not considered). A) Comparison of contact energies per residue for B42 and vMIP-I. B) Comparison of contact energies per residue for N73 and vMIP-II. Secondary structure elements of the templates are indicated below as arrows for β-strand and cylinders for α-helix.
(TIF)

Figure S3 **Contact energy correlation analysis.** Correlation of contact energies pairs of the putative chemokine models of B42 and N73 with their corresponding template reference models. A) B42 model vs. reference model vMIP-I (R^2^ = 0.63). B) N73 model vs. reference model vMIP-II (R^2^ = 0.41). C) Reference model vMIP-I vs. X-ray structure of CXCL8 mutant (R^2^ = 0.43). D) Reference model vMIP-II vs. X-ray structure of CXCL8 mutant (R^2^ = 0.51).
(TIF)

Figure S4 **Ensembl annotations of B42 and N73.** A) B42. Coding regions on exons are indicated by filled boxes. The open reading frame is located on chromosome 19q13.41. 1) Ensembl release 54, 2) Ensembl release 55 and 3) Ensembl release 61. The chromosomal location of B42 in Ensembl release 62 is highlighted in green and labeled as ZNF578 pseudogene with surrounding genes in the upper right, and the annotated exon structure is shown below. B) N73. The coding region on exon 1 is indicated by a filled box. The open reading frame is located on chromosome 8q24.3. The chromosomal location in Ensembl release 61 with surrounding genes is shown in the upper right, and the exon structure is shown below.
(TIF)

Table S1 **Existing prediction tools applicable to chemokine identification in comparison with our 3D profile-based methodology (3D-CKpred).** *SP/TM*: prediction of signal peptides (SP) and transmembrane regions (TM); *Batch*: processing of many sequences in one batch is supported (‘+’) or not (‘−’); *Seq/Str:* sequence-based (seq) or a structure-based (str) approach; *diS*: disulfide bond prediction based on 3D arrangement of cysteine residues.
(DOC)

Table S2 **Ten best candidate proteins selected for atomic 3D model building and their respective threading results with the** ***pdb95 fold library*** **.** The top (1-2) chemokine threading hit obtained for each query protein is shown including the PDB template structure used for their respective molecular modelling. The ID of the proteins corresponding to the four best 3D models selected for further refinement and analysis are displayed in bold. *ID*: Protein name; *UniProt*: UniProt identifier; *Seq*: sequence region selected; *Rank*: Top 1 or 2 ranking of the chemokine fold in the threading control experiments across fold space (*pdb95 fold library*); *Thx*: ProHit threading Index; *%ID*: percentage of sequence identity between query and template; *Template*: PDB template; *PDB*: template identifier in the Protein Data Bank; *Chain*: template chain; *fl*: fold length; *pl*: path length; *diS*: sequence number of cysteine residue pairs with possibilities of disulfide bond formation.
(DOC)

Table S3 **Transcription factor binding sites found in the promoter region of the human and orangutan B42 genes.** Overview of transcription factor binding sites (TFBS) found in the promoter region of the human and orangutan B42 genes with the corresponding sequence pattern, its relative position to the transcription start of the B42 gene, and chemokine genes known to present those binding sites.
(DOC)

Table S4 **Fold recognition results obtained for N73 with the Chemokine fold library.** Top 5 scoring structures are shown. *Rank*: Rank of the template chemokine within all chemokine structures in the fold library; *Thx*: threading index; *%ID*: percentage of amino acid sequence identity between query and template; *pl*: alignment path length; *fl*: template fold length; *PDB*: template identifier in the Protein Data Bank; *chain*: template chain; *description*: description of PDB template; *UniProt*: UniProt identifier; *Rec. agonist*: Chemokine receptors for which the template protein is an agonist; *Rec. antagonist*: Chemokine receptors for which the template protein is an antagonist.
(DOC)

Table S5 **Fold recognition results obtained for N73 with the pdb95 fold library.** Top 5 SCOP folds are shown. *Conf*: Confidence level (HC, high confidence; FP, false positive); *secr*: secretion; *sse*: all secondary structure elements present in alignment; *gap*: gap length >10 amino acids; *fcov*: fold coverage based on ratio of fold length/path length; *Thx*: threading index; *%ID*: percentage of amino acid sequence identity between query and template; *pl*: alignment path length; *fl*: template fold length; *SCOP*: SCOP family identifier; *PDB*: template identifier in the Protein Data Bank; *chain*: template chain; *% cover*.: percentage of domain coverage; *description*: description of PDB template; *Pfam*: Pfam description; *UniProt*: template UniProt identifier.
(DOC)

Table S6 **Transcription factor binding sites found in the promoter region of the human N73 gene.** Overview of transcription factor binding sites (TFBS) found in the promoter region of the human N73 gene with the corresponding sequence pattern, its relative position to the transcription start of the N73 gene, and chemokine genes known to present those binding sites.
(DOC)

Table S7 **Threading results of CXCL17 with the** ***pdb95 fold library*** **.** Only best template per SCOP fold is shown. The chemokine fold is highlighted in bold. Abbreviations are the same as in Table S5. Results obtained in CXCL identification [10] are shown below for comparison.
(DOC)

Text S1 (DOC)
