# Supplementary figures and images for: 3D Profile-Based Approach to Proteome-Wide Discovery of Novel Human Chemokines
Source: PLoS One. 2012 May 7;7(5):e36151. doi: 10.1371/journal.pone.0036151 (PMC3346806; doi:10.1371/journal.pone.0036151)

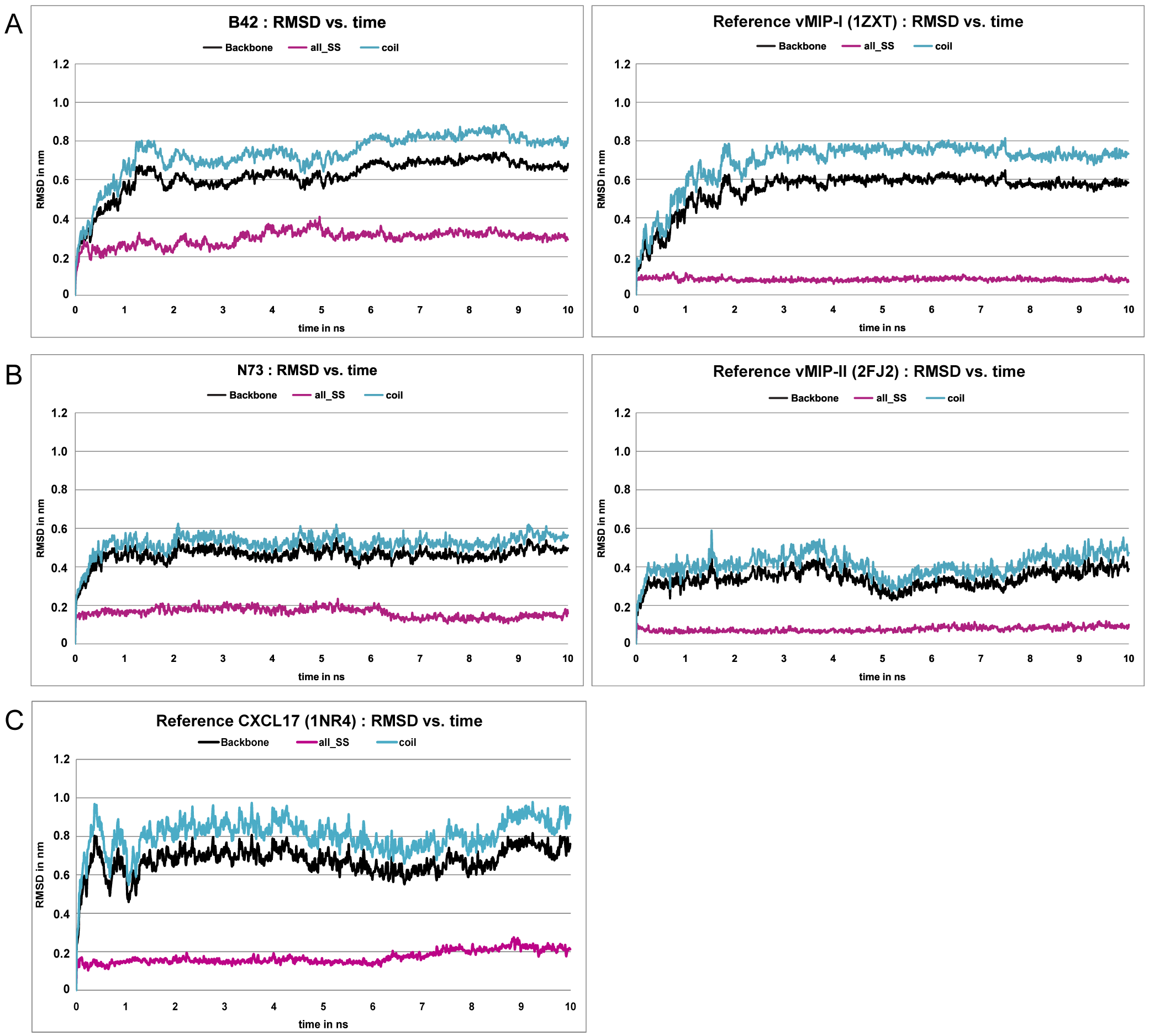

Supplement: Figure S1 — Results of MD simulations for chemokine-like models of B42, N73, and the reference models of vMIP-I, vMIP-II and CXCL17. A) RMSD along the MD simulation of the chemokine-like B42 model (left) and vMIP-I model (right). B) RMSD along the MD simulation of the chemokine-like N73 model (left), and vMIP-II model (right). C) RMSD along the MD simulation of the CXCL17 model. Black lines correspond to backbone, margenta to secondary structure elements, and blue to coil regions. (TIF) [file pone.0036151.s001.tif]

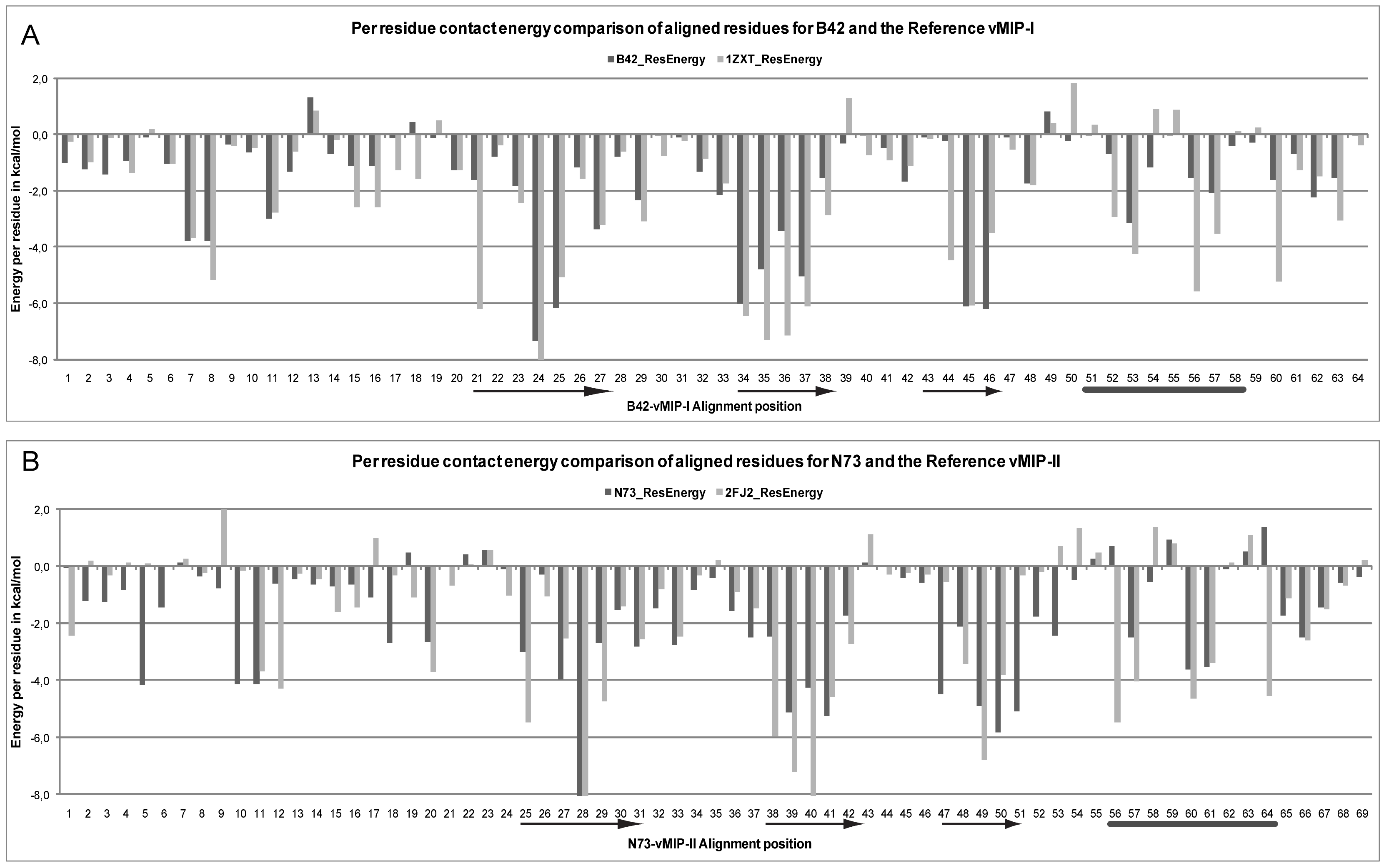

Supplement: Figure S2 — Contact energy decomposition plots. Contact energies of the residues in the putative chemokine models are compared to the corresponding residues in their template structures (gaps are not considered). A) Comparison of contact energies per residue for B42 and vMIP-I. B) Comparison of contact energies per residue for N73 and vMIP-II. Secondary structure elements of the templates are indicated below as arrows for β-strand and cylinders for α-helix. (TIF) [file pone.0036151.s002.tif]

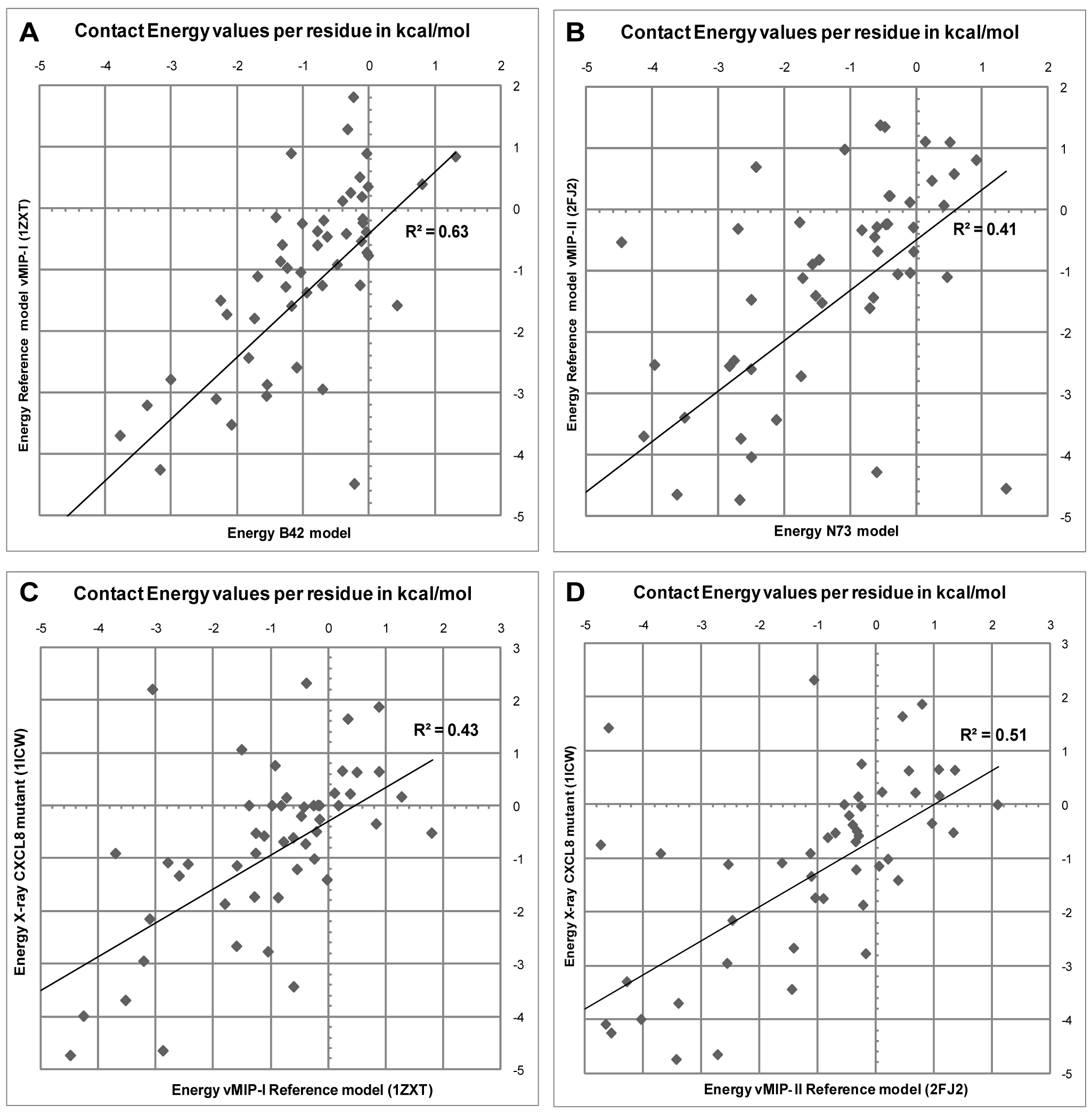

Supplement: Figure S3 — Contact energy correlation analysis. Correlation of contact energies pairs of the putative chemokine models of B42 and N73 with their corresponding template reference models. A) B42 model vs. reference model vMIP-I (R2 = 0.63). B) N73 model vs. reference model vMIP-II (R2 = 0.41). C) Reference model vMIP-I vs. X-ray structure of CXCL8 mutant (R2 = 0.43). D) Reference model vMIP-II vs. X-ray structure of CXCL8 mutant (R2 = 0.51). (TIF) [file pone.0036151.s003.tif]

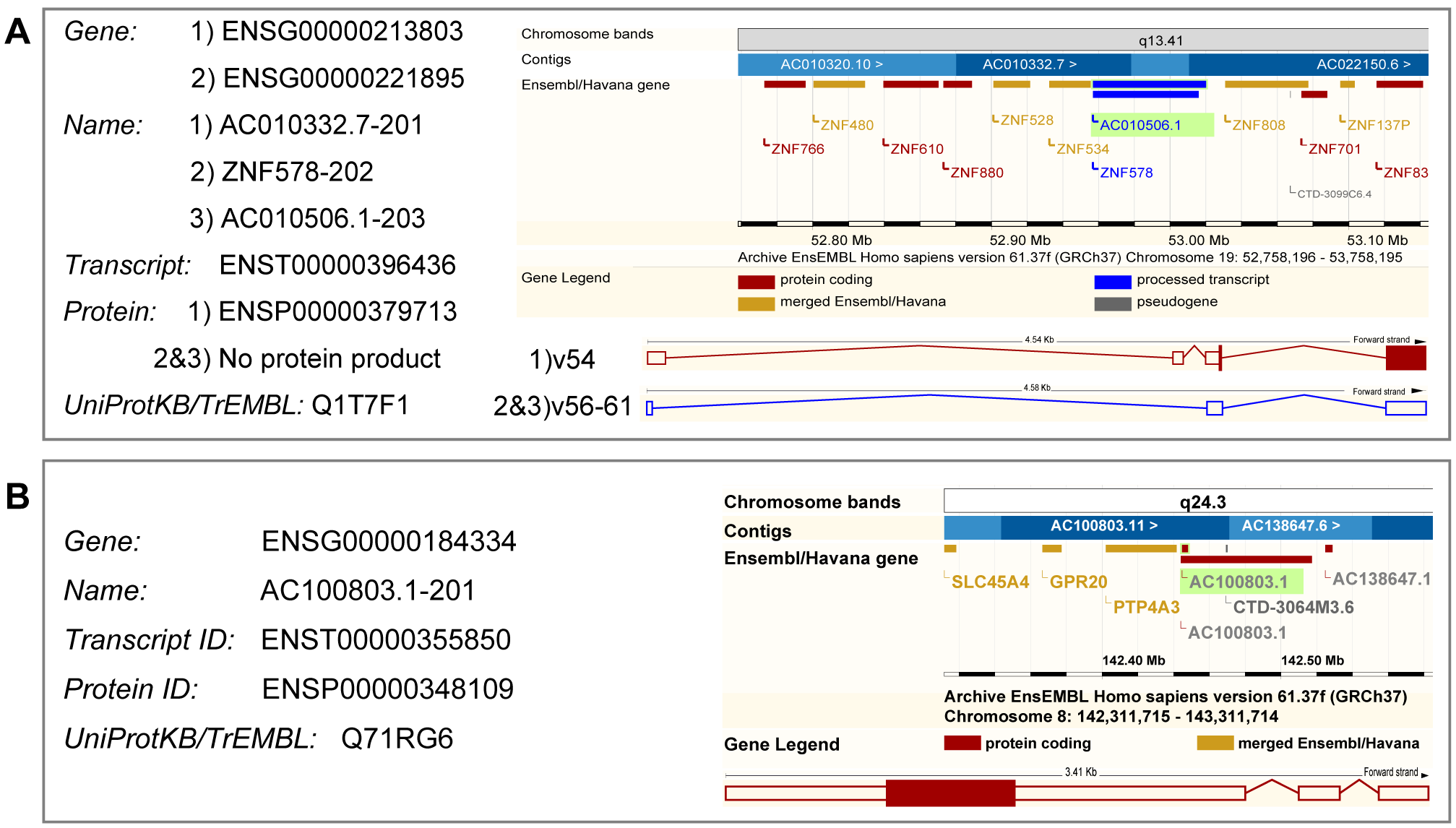

Supplement: Figure S4 — Ensembl annotations of B42 and N73. A) B42. Coding regions on exons are indicated by filled boxes. The open reading frame is located on chromosome 19q13.41. 1) Ensembl release 54, 2) Ensembl release 55 and 3) Ensembl release 61. The chromosomal location of B42 in Ensembl release 62 is highlighted in green and labeled as ZNF578 pseudogene with surrounding genes in the upper right, and the annotated exon structure is shown below. B) N73. The coding region on exon 1 is indicated by a filled box. The open reading frame is located on chromosome 8q24.3. The chromosomal location in Ensembl release 61 with surrounding genes is shown in the upper right, and the exon structure is shown below. (TIF) [file pone.0036151.s004.tif]
