# Supplementary material for: 3D Profile-Based Approach to Proteome-Wide Discovery of Novel Human Chemokines
Source: PLoS One. 2012 May 7;7(5):e36151. doi: 10.1371/journal.pone.0036151 (PMC3346806; doi:10.1371/journal.pone.0036151)
Supplement: Table S1 — Existing prediction tools applicable to chemokine identification in comparison with our 3D profile-based methodology (3D-CKpred). SP/TM: prediction of signal peptides (SP) and transmembrane regions (TM); Batch: processing of many sequences in one batch is supported (‘+’) or not (‘−’); Seq/Str: sequence-based (seq) or a structure-based (str) approach; diS: disulfide bond prediction based on 3D arrangement of cysteine residues. (DOC) [file pone.0036151.s005.doc]

**Table S1: Existing prediction tools applicable to chemokine identification in comparison with our 3D profile-based methodology (3D-CKpred).**

| **Method** | **Description** | **SP/TM** | **Batch** | **Seq/Str** | **diS** |
| --- | --- | --- | --- | --- | --- |
| **SMART** | SCY Intercrine alpha family (small cytokine C-X-C) (SM00199) | +/+ | + | seq | - |
| **Pfam HMM** | Small cytokines (intercrine/chemokine), interleukin-8 like (PF00048) | +/+ | + | seq | - |
| **PROSITE pattern** | Chemokine C-C subfamily signature (PS00472)  Chemokine C-x-C subfamily signature (PS00471) | -/- | + | seq | - |
| **ChemoPred** | Machine learning technique to identify novel chemokines | -/- | - | seq | - |
| **3D-CKpred** | Threading combined with structure-based disulfide bond validation | +/+ | + | str | + |

*SP/TM*: prediction of signal peptides (SP) and transmembrane regions (TM); *Batch*: processing of many sequences in one batch is supported (‘+’) or not (‘-’); *Seq/Str:* sequence-based (seq) or a structure-based (str) approach; *diS*: disulfide bond prediction based on 3D arrangement of cysteine residues.
