# Supplementary material for: 3D Profile-Based Approach to Proteome-Wide Discovery of Novel Human Chemokines
Source: PLoS One. 2012 May 7;7(5):e36151. doi: 10.1371/journal.pone.0036151 (PMC3346806; doi:10.1371/journal.pone.0036151)
Supplement: Table S2 — Ten best candidate proteins selected for atomic 3D model building and their respective threading results with the pdb95 fold library . The top (1-2) chemokine threading hit obtained for each query protein is shown including the PDB template structure used for their respective molecular modelling. The ID of the proteins corresponding to the four best 3D models selected for further refinement and analysis are displayed in bold. ID: Protein name; UniProt: UniProt identifier; Seq: sequence region selected; Rank: Top 1 or 2 ranking of the chemokine fold in the threading control experiments across fold space (pdb95 fold library); Thx: ProHit threading Index; %ID: percentage of sequence identity between query and template; Template: PDB template; PDB: template identifier in the Protein Data Bank; Chain: template chain; fl: fold length; pl: path length; diS: sequence number of cysteine residue pairs with possibilities of disulfide bond formation. (DOC) [file pone.0036151.s006.doc]

**Table S2: Ten best candidate proteins selected for atomic 3D model building and their respective threading results with the *pdb95 fold library*.**

| **ID** | **UniProt** | **Seq** | **Rank** | **Thx** | **%ID** | **Template** | **PDB** | **Chain** | **pl** | **fl** | **diS** |
| --- | --- | --- | --- | --- | --- | --- | --- | --- | --- | --- | --- |
| L07 | Q8WYU1_HUMAN | 0-165 | 1 | 49.0 | 23.2 | CCL3 | 1B50 | B | 69 | 69 | (69, 91), (69, 103), (69, 105), (69, 111), (70, 91), (70, 103), (70, 105), (70, 110), (70, 111), (70, 128), (91, 103), (91, 105), (91, 110), (91, 111), (91, 128), (110, 144) |
| **B42** | Q1T7F1_HUMAN | 0-81 | 1 | 45.6 | 22.6 | vMIP-I | 1ZXT | A | 62 | 69 | (7, 29), (7, 45), (8, 29), (8, 45), (11, 45) |
| B58 | Q8IUQ3_HUMAN | 103-172 | 1 | 35.3 | 19.3 | vMIP-II | 1CM9 | A | 57 | 68 | (8, 47), (8, 57), (17, 57) |
| A93 | CX028_HUMAN | 0-62 | 1 | 30.6 | 17.7 | CXCL12 | 1A15 | B | 51 | 57 | (15, 57) |
| **G19** | Q96KT8_HUMAN | 0-120 | 2 | 40.8 | 22.9 | vMIP-II | 1HHV | A | 70 | 73 | (12, 20) |
| H86 | Q96RX8_HUMAN | 0-132 | 2 | 34.0 | 21.5 | CXCL4 | 1F9S | C | 65 | 62 | (67, 86), (67, 88), (67, 119) |
| **N73** | YH006_HUMAN | 0-208 | 2 | 33.7 | 23.2 | vMIP-II | 2FJ2 | D | 69 | 67 | (72, 78), (72, 98), (72, 125), (77, 98), (77, 125), (78, 98), (78, 125), (78, 127), (98, 125) |
| **L32** | YA026_HUMAN | 0-169 | 2 | 33.2 | 21.9 | CCL14 | 2Q8T | C | 73 | 65 | (68, 112), (69, 112) |
| G71 | CK076_HUMAN | 0-123 | 2 | 32.2 | 23.7 | CCL2 | 1DOL | A | 76 | 71 | (36, 87), (36, 88), (43, 88) |
| D56 | Q9NQU3_HUMAN | 0-93 | 2 | 30.8 | 17.4 | vMIP-I | 1ZXT | C | 69 | 69 | (25, 70), (25, 71), (71, 77) |

The top (1-2) chemokine threading hit obtained for each query protein is shown including the PDB template structure used for their respective molecular modelling. The ID of the proteins corresponding to the four best 3D models selected for further refinement and analysis are displayed in bold. *ID*: Protein name; *UniProt*: UniProt identifier; *Seq*: sequence region selected; *Rank*: Top 1 or 2 ranking of the chemokine fold in the threading control experiments across fold space (*pdb95 fold library*); *Thx*: ProHit threading Index; *%ID*: percentage of sequence identity between query and template; *Template*: PDB template; *PDB*: template identifier in the Protein Data Bank; *Chain*: template chain; *fl*: fold length; *pl*: path length; *diS*: sequence number of cysteine residue pairs with possibilities of disulfide bond formation.
