# Supplementary material for: 3D Profile-Based Approach to Proteome-Wide Discovery of Novel Human Chemokines
Source: PLoS One. 2012 May 7;7(5):e36151. doi: 10.1371/journal.pone.0036151 (PMC3346806; doi:10.1371/journal.pone.0036151)
Supplement: Table S3 — Transcription factor binding sites found in the promoter region of the human and orangutan B42 genes. Overview of transcription factor binding sites (TFBS) found in the promoter region of the human and orangutan B42 genes with the corresponding sequence pattern, its relative position to the transcription start of the B42 gene, and chemokine genes known to present those binding sites. (DOC) [file pone.0036151.s007.doc]

**Table S3: Transcription factor binding sites found in the promoter region of the human and orangutan B42 genes.**

| **Transcription factor binding site (TFBS)** | **Sequence Pattern** | **Position relative to transcription start in human / orangutan B42** | **Chemokine genes presenting this TFBS** |
| --- | --- | --- | --- |
| NF-IL6 (CCAAT/enhancer binding prot.) | CCAAT | -314/ -315 | CCL3, CCL4, CXCL8 |
| NF-kappaB | Different pattern | 6 different BS | CXCL2, CXCL8, CCL5, CXCL10 |
| AP1 (C-Jun) | TGATGTCA & others | -756/ -765 and -763/ -654 | CCL3, CCL4, CXCL8 |
| INF-1 | AACTCA | -90/ -91 | CCL3, CCL4, CXCL2 |
| CK1: motif | GAGATTCCCAT | +2774 / +1561  Both 1^st^ intron with 1 mismatch | +63 nucleotides after transcription start in CCL3 and +85 in CCL4 |

Overview of transcription factor binding sites (TFBS) found in the promoter region of the human and orangutan B42 genes with the corresponding sequence pattern, its relative position to the transcription start of the B42 gene, and chemokine genes known to present those binding sites.
