# Supplementary material for: 3D Profile-Based Approach to Proteome-Wide Discovery of Novel Human Chemokines
Source: PLoS One. 2012 May 7;7(5):e36151. doi: 10.1371/journal.pone.0036151 (PMC3346806; doi:10.1371/journal.pone.0036151)
Supplement: Table S4 — Fold recognition results obtained for N73 with the Chemokine fold library. Top 5 scoring structures are shown. Rank: Rank of the template chemokine within all chemokine structures in the fold library; Thx: threading index; %ID: percentage of amino acid sequence identity between query and template; pl: alignment path length; fl: template fold length; PDB: template identifier in the Protein Data Bank; chain: template chain; description: description of PDB template; UniProt: UniProt identifier; Rec. agonist: Chemokine receptors for which the template protein is an agonist; Rec. antagonist: Chemokine receptors for which the template protein is an antagonist. (DOC) [file pone.0036151.s008.doc]

**Table S4: Fold recognition results obtained for N73 with the Chemokine fold library.**

| **Rank** | **Thx** | **%ID** | **CK Template** | **PDB** | **Chain** | **pl** | **fl** | **UniProt** | **Ragonist** | **Rantagonist** |
| --- | --- | --- | --- | --- | --- | --- | --- | --- | --- | --- |
| 1 | 36.3 | 23.2 | vMIP-II | 2FJ2 | 2FJ2 | 69 | 71 | VMI2_HHV8 | CCR3 | CCR1,2,5, CXCR4, XCR1, CX3CR1 |
| 2 | 23.4 | 18.7 | vMIP-I | 1ZXT | 1ZXT | 75 | 76 | Q76RJ0_HHV8 | CCR8, part. CCR3 | part. CCR5 |
| 3 | 19.6 | 21.0 | MIP-3 alpha | 1M8A | 1M8A | 62 | 70 | CCL20_HUMAN | CCR6 |  |
| 4 | 17.8 | 11.8 | CCL3 | 2X6G | 2X6G | 68 | 61 | CCL3_HUMAN | CCR1, CCR5 | - |
| 5 | 17.6 | 18.2 | GRO-beta(5-73) | 1QNK | 1QNK | 66 | 69 | CXCL2_HUMAN |  |  |

Top 5 scoring structures are shown. *Rank*: Rank of the template chemokine within all chemokine structures in the fold library; *Thx*: threading index; *%ID*: percentage of amino acid sequence identity between query and template; *pl*: alignment path length; *fl*: template fold length; *PDB*: template identifier in the Protein Data Bank; *chain*: template chain; *description*: description of PDB template; *UniProt*: UniProt identifier; *Rec. agonist*: Chemokine receptors for which the template protein is an agonist; *Rec. antagonist*: Chemokine receptors for which the template protein is an antagonist.
