# Supplementary material for: 3D Profile-Based Approach to Proteome-Wide Discovery of Novel Human Chemokines
Source: PLoS One. 2012 May 7;7(5):e36151. doi: 10.1371/journal.pone.0036151 (PMC3346806; doi:10.1371/journal.pone.0036151)
Supplement: Table S5 — Fold recognition results obtained for N73 with the pdb95 fold library. Top 5 SCOP folds are shown. Conf: Confidence level (HC, high confidence; FP, false positive); secr: secretion; sse: all secondary structure elements present in alignment; gap: gap length >10 amino acids; fcov: fold coverage based on ratio of fold length/path length; Thx: threading index; %ID: percentage of amino acid sequence identity between query and template; pl: alignment path length; fl: template fold length; SCOP: SCOP family identifier; PDB: template identifier in the Protein Data Bank; chain: template chain; % cover.: percentage of domain coverage; description: description of PDB template; Pfam: Pfam description; UniProt: template UniProt identifier. (DOC) [file pone.0036151.s009.doc]

**Table S5: Fold recognition results obtained for N73 with the pdb95 fold library.**

| **Rank** | **Conf** | **secr** | **sse** | **gap** | **fcov** | **Thx** | **%ID** | **SCOP** | **%Cov** | **Template** | **PDB** | **Chain** | **pl** | **fl** | **UniProt** | **Pfam** |
| --- | --- | --- | --- | --- | --- | --- | --- | --- | --- | --- | --- | --- | --- | --- | --- | --- |
| 1 | FP | - | - | **+** | **+** | 41.4 | 18.4 | b.47.1.3 | 84 | Chimera of non-structural protein 2B and 3 | 2GGV | B | 136 | 169 | - | Peptidase_S7 |
| 2 | FP | - | - | - | - | 40.7 | 23.8 | d.24.1.7 | 98 | Aflatoxin biosynthesis polyketide synthase | 3HRQ | B | 122 | 319 | PKSL1_ASPPA | - |
| 3 | FP | - | + | - | - | 40.6 | 20.2 | c.23.1.1 | 95 | Response regulator/HD domain protein | 3HV2 | A | 104 | 136 | Q4K707_PSEF5 | Response_reg |
| 4 | FP | - | - | **+** | - | 36.5 | 16.1 | b.42.2.1 | 98 | Agglutinin II (SNAV) | 3CA6 | A | 124 | 257 | NIGB_SAMNI | Ricin_B_lectin |
| 5 | **HC** | **+** | **+** | **+** | **+** | **36.3** | **23.2** | **d.9.1.1** | **100** | **vMIP-II** | **2FJ2** | **D** | **69** | **67** | **VMI2_HHV8P** | **IL8** |

Top 5 SCOP folds are shown. *Conf*: Confidence level (HC, high confidence; FP, false positive); *secr*: secretion; *sse*: all secondary structure elements present in alignment; *gap*: gap length > 10 amino acids; *fcov*: fold coverage based on ratio of fold length/path length; *Thx*: threading index; *%ID*: percentage of amino acid sequence identity between query and template; *pl*: alignment path length; *fl*: template fold length; *SCOP*: SCOP family identifier; *PDB*: template identifier in the Protein Data Bank; *chain*: template chain; *% cover*.: percentage of domain coverage; *description*: description of PDB template; *Pfam*: Pfam description; *UniProt*: template UniProt identifier.
