# Supplementary material for: 3D Profile-Based Approach to Proteome-Wide Discovery of Novel Human Chemokines
Source: PLoS One. 2012 May 7;7(5):e36151. doi: 10.1371/journal.pone.0036151 (PMC3346806; doi:10.1371/journal.pone.0036151)
Supplement: Table S6 — Transcription factor binding sites found in the promoter region of the human N73 gene. Overview of transcription factor binding sites (TFBS) found in the promoter region of the human N73 gene with the corresponding sequence pattern, its relative position to the transcription start of the N73 gene, and chemokine genes known to present those binding sites. (DOC) [file pone.0036151.s010.doc]

**Table S6: Transcription factor binding sites found in the promoter region of the human N73 gene.**

| **Transcription factor binding site (TFBS)** | **Sequence Pattern** | **Position relative to transcription start in human** | **Chemokine genes presenting this TFBS** |
| --- | --- | --- | --- |
| NF-IL6 (CCAAT/enhancer binding prot.) | CCAAT | 1x in 3^rd^ exon | CCL3, CCL4, CXCL8 |
| NF-kappaB | GGGGAAGGGCCA | -217 | CXCL2, CXCL8, CCL5, CXCL10 |
| AP1 (C-Jun) | TGACTCCAG | -357 | CCL3, CCL4, CXCL8 |

Overview of transcription factor binding sites (TFBS) found in the promoter region of the human N73 gene with the corresponding sequence pattern, its relative position to the transcription start of the N73 gene, and chemokine genes known to present those binding sites.
