# Supplementary material for: 3D Profile-Based Approach to Proteome-Wide Discovery of Novel Human Chemokines
Source: PLoS One. 2012 May 7;7(5):e36151. doi: 10.1371/journal.pone.0036151 (PMC3346806; doi:10.1371/journal.pone.0036151)
Supplement: Table S7 — Threading results of CXCL17 with the pdb95 fold library . Only best template per SCOP fold is shown. The chemokine fold is highlighted in bold. Abbreviations are the same as in Table S5. Results obtained in CXCL identification [10] are shown below for comparison. (DOC) [file pone.0036151.s011.doc]

**Table S7: Threading results of CXCL17 with the *pdb95 fold library*.**

| **Rank** | **Conf** | **fcov** | **Thx** | **%ID** | **SCOP** | **% Cov** | **Template** | **PDB** | **Chain** | **pl** | **fl** | **UniProt** | **Pfam** |
| --- | --- | --- | --- | --- | --- | --- | --- | --- | --- | --- | --- | --- | --- |
| 1 | HC | + | 35.8 | 23.0 | g.39.1.2 | 99 | Hepatocyte Nuclear Factor 4-alpha, DNA binding domain | 3CBB | B | 74 | 78 | P41235 | zf-C4 |
| 2 | HC | + | 31.0 | 18.8 | d.93.1.1 | 91 | SAP SH2 domain | 1D1Z | D | 85 | 101 | O60880 | SH2 |
| 3 | HC | + | 28.3 | 20.3 | d.52.9.1 | 90 | CzrB protein | 3BYP | A | 74 | 82 | Q8VLX7 | Cation_efflux |
| 4 | FP | - | 28.2 | 25.5 | b.36.1.1 | 71 | Regulator of G-protein signalling 3 isoform 1 | 2F5Y | A | 55 | 82 | P49796 | PDZ |
| **5** | **HC** | **+** | **27.9** | **21.4** | **d.9.1.1** | **100** | **CCL17: Thymus and activation-regulated chemokine** | **1NR4** | **D** | **70** | **63** | **Q92583** | **IL8** |
| 6 | HC | + | 27.7 | 19.0 | d.50.1.1 | 100 | RISC-loading complex subunit TARBP2/RNA complex | 3ADL | A | 79 | 76 | Q15633 | dsrm |
| 7 | HC | + | 27.1 | 18.6 | g.7.1.3 | 99 | Bone morphogenetic protein receptor type-2 | 2HLR | A | 70 | 67 | Q91WY9 | Activin_recp |
| 8 | FP | - | 26.4 | 17.4 | d.113.1.1 | 75 | MUTT/NUDIX Family Protein | 2W4E | A | 86 | 137 | Q9RSC1 | NUDIX |
| 9 | FP | - | 26.1 | 20.8 | g.3.11.1 | 93 | Urokinase-type plasminogen activator | 2I9A | D | 48 | 124 | P00749 | Kringle |
| 10 | FP | - | 26.1 | 22.2 | g.18.1.1 | 89 | Fiber 36.1 kDa protein, Membrane cofactor protein | 3O8E | D | 54 | 248 | P15529 | Sushi |
| 11 | FP | - | 26.1 | 14.3 | d.37.1.1 | 75 | Uncharacterized protein CT1051 | 3GBY | A | 91 | 127 | Q8KDJ9 | CBS |
| 12 | HC | + | 25.7 | 14.3 | a.3.1.1 | 100 | Cytochrome Oxidase Subunit II | 1W2L | A | 77 | 97 | Q9F3S9 | Cytochrom_C |
| 13 | FP | - | 25.5 | 22.2 | a.4.5.32 | 83 | Transcriptional Regulatory Protein | 2VC0 | A | 54 | 147 | P96896 | AsnC_trans_reg |
| 14 | FP | - | 25.5 | 24.5 | g.44.1.2 | 78 | E3 Ubiquitin-protein Ligase MDM2, MDM4 | 2VJF | A | 49 | 64 | Q00987 | - |
| **-** | **HC** | **+** | **25.3** | **14.3** | **d.9.1.1** | **100** | **CXCL8: Interleukin-8** | **1ICW** | **B** | **70** | **66** | **P10145** | **IL8** |

Only best template per SCOP fold is shown. The chemokine fold is highlighted in bold. Abbreviations are the same as in Table S5. Results obtained in CXCL identification [10] are shown below for comparison.
