# Supplementary material for: 3D Profile-Based Approach to Proteome-Wide Discovery of Novel Human Chemokines
Source: PLoS One. 2012 May 7;7(5):e36151. doi: 10.1371/journal.pone.0036151 (PMC3346806; doi:10.1371/journal.pone.0036151)
Supplement: Text S1 — (DOC) [file pone.0036151.s012.doc]

Supporting Text

I. Existing methodology for chemokine identification in comparison with our 3D chemokine profile-based methodology.

The methodology developed and applied in this work (referred as 3D-CKpred) is compared in Table S1 to other existing prediction tools applicable to chemokine identification: SMART , Pfam HMM PROSITE pattern ChemoPred . Our methodology uses experimentally known chemokine structures as basis for structure prediction by fold recognition methods in combination with an approach to detect possible disulfide bonds in the predicted 3D folds. As most other methods, 3D-CKpred includes sequence-based techniques to predict signal peptides and transmembrane regions. Like Pfam, SMART and PROSITE, it also provides the possibility of processing several protein sequences in a batch-processing mode. In particular, our approach automatically recognizes remote homologs of chemokine signatures by identifying possible disulfide bonds at different positions in sequence and in structure through fold prediction.

II. Computational discovery and characterization of chemokine-like protein N73

Fitting of the N73 sequence into the 3D chemokine-like fold profile

Table S4 shows the best five protein hits resulting of threading the N73 protein sequence against the *Chemokine fold library,* which includes all currently available structural information about the chemokine fold. The top 2 ranking alignments are found with Kaposi’s sarcoma-associated herpesvirus encoded vMIP-II and vMIP-I with 23.2% and 18.7% sequence identity, respectively. As mentioned before, both proteins can block HIV entry through the chemokine receptors CCR3 and CCR5 . CCL20 (rank 3) is found as best scoring human chemokine, followed by the human chemokine CCL3 (rank 4) and CXCL2 (rank 5). CCL20 is the only natural ligand of CCR6 that has important functions in the defense response to bacteria by attraction of natural killer and memory T cells to sites of inflammation.

Fitting of the N73 sequence into the chemokine-like fold across fold space

When N73 is threaded against the *pdb95 fold library*, which contains representatives of all protein folds currently available in the PDB, the IL8-like chemokine fold appears as best ranked hit of high confidence with the template v-MIP-II (Table S5). We applied our filtering criteria based on full fold coverage (‘fcov’ column), inclusion of secondary structure motifs (‘sse’) and low gap content (‘gap’) to establish prediction confidence levels to the hit list (HC = high confidence; FP = false positives; see methods for details). In addition to the filter criteria applied previously for B42, we considered the fact that the right template for N73 should be a secreted protein due to the presence of a secretory signal peptide in the N73 sequence (see above). Therefore, only secreted folds (indicated with + in ‘secr’ column) were considered as templates for modeling of N73. In the alignment of the N73 sequence to the v-MIP-II structure, we could observe the possibility of formation of three disulfide bonds (see below in modeling details), which reinforces the finding that N73 may favor to fold into an IL8-like structure over all other known folds. These results were strongly indicative of the protein N73 being a possible remote structural homologue of the IL8-like chemokine family.

Modeling N73 into a functional IL8-like chemokine structure

The top threading hit vMIP-II (PDB: 2FJ2, resolution: 2.3 Å; Table S4) was used as structural template to model N73 in an IL8-like chemokine structure (Figure 3A). Although N73 and vMIP-II share low sequence identity (23.2 %), once N73 sequence is folded into the IL8-like structure, it can be seen that structurally relevant residues are perfectly disposed in three-dimensions to form a functional fold: i) cysteines distributed along the N73 sequence are coming together in 3D to form disulfides, which are disposed in 3D in non-canonical positions; ii) relevant amino acids forming the core of the fold are well conserved. The resulting 3D model of N73 offered two different possibilities to form disulfide bonds equivalent to those in the template structure vMIP-II: i) two disulfide bonds between C54 - C75 and C55 - C102, which would correspond to the CC-motif arrangement of the template; ii) three disulfide bonds between C49 - C75, C54 - C102 and C55 - C104. Figure 3B shows the detailed 3D model of N73 as an IL8-like chemokine structure with 3 disulfide bonds in comparison to the vMIP-II structure used as template to model it.

The 3D model of N73 was energy refined in a 10 ns molecular dynamics (MD) simulation. A model of vMIP-II and CXCL17 were used as references for analysis as in the case of B42 (see methods for details). In general, the reference model of vMIP-II and N73 showed similar behavior during the MD trajectory (Figure S1 B). Similar to the reference, the RMSD values of N73 converge over time, indicating that the overall IL8-like chemokine fold stays compact along the simulation. Only the insertion in N73 connecting the 2^nd^ and 3^rd^ sheet is flexible and packs more tightly to the protein core during the simulation, which results in a higher RMSD of coil regions. These results are also comparable to the results obtained for our second reference model, the CXCL17 (Figure S1 C). Our observations further support the prediction that the N73 sequence may adopt a compact IL8-like chemokine fold.

In order to quantify the residue energetic contributions in the N73 chemokine-like model, we calculated contact energies per residue and compared them to those of the vMIP-II model. The contact energy decomposition plot for N73 compared to vMIP-II shows a general agreement in the energetic contributions of the corresponding residues. In particular, it can be observed that the secondary structure regions and the core of the protein present a good agreement of the contact energies, indicating that the packing of the core of N73 is comparable to the packing of the vMIP-II template (Figure S2 B). We calculated the coefficient of determination (R²) from the contact energy pairs of the N73 model and the reference model of vMIP-II and obtained an R² value of 0.41 (Figure S3 B). We also did this analysis for the vMIP-II model with reference to a mutant of CXCL8 (PDB: 1ICW), and obtained an R² value of 0.51 (Figure S3 D). We selected this CXCL8 mutant as reference, because it has one disulfide bond at a non-canonical position when compared to the rest of the chemokine protein family, and it has low sequence identity to vMIP-II (19 %). The R² value obtained for N73 is slightly lower than for the chemokine control but still in the same range as the second chemokine control that was used for vMIP-I and CXCL8 (Figure S3 C), which is indicative of a good packing of the core in the N73 model compared to known chemokines and gives high confidence to its predicted structural resemblance towards the IL8-like chemokine fold. The possibility that N73 could be a remote homologue of the chemokine protein family was further explored by searching for the presence of chemokine-like features in its protein and gene sequence.

N73 protein sequence and gene analysis

In the N73 protein sequence, we identified a signal peptide that is cleaved at sequence position 23 from the N73 precursor sequence (Figure 3C) resulting in the mature N73 protein with 8 cysteines (Figure 3 A and B). No transmembrane region was predicted. Also WoLF PSORT predicted extracellular location for N73 with a high confidence (25 out of the maximal possible 32 nearest neighbors are extracellular localized). The ELM server was queried with the N73 precursor sequence using the extracellular filter and predicted two low complexity regions (LCR) (21 amino acids long at the N-terminus and 19 amino acids at the C-terminus indicated in Figure 3C). In addition, an N-glycosylation site (N_29_) and the linear motif PSAR in residues 147-150 for a glycosaminoglycan attachment at a serine were identified, both being common chemokine features. The GlobPlot program predicted several disordered regions (Figure 3C).

Another interesting feature of N73 is the RGD motif found at positions 198-200 (Figure 3C), as this is a receptor binding motif recognized by different members of the integrin family. To be functional, the RGD motif has to lie on an exposed flexible loop , which is also the case for N73. RGD motifs in proteins of the extracellular matrix are used by integrins to link the intracellular cytoskeleton of cells with the extracellular matrix. Without attachment to the extracellular matrix, cells normally undergo apoptosis ('anoikis') . Small soluble polypeptides containing the RGD motif, such as chemokines, inhibit cell attachment and consequently induce apoptosis of the integrin presenting cells . This makes RGD containing peptides and mimetics potential therapeutic agents against tumor-induced angiogenesis, inflammation and cancer metastasis. Thus, it might be possible that the soluble secreted N73 protein can perform similar functions through integrin interaction with its RGD motif.

N73 is conserved across different primate species. Ensembl’s automatic annotation pipeline (release 61) identified one close ortholog in gorilla (Ensembl release 61: [ENSGGOG00000010606](http://www.ensembl.org/Gorilla_gorilla/Gene/Summary?g=ENSGGOG00000010606;db=core)) sharing 96% protein sequence identity and another in bushbaby (Ensembl release 61: [ENSOGAG00000013938](http://www.ensembl.org/Otolemur_garnettii/Gene/Summary?g=ENSOGAG00000013938;db=core)) sharing 45% protein sequence identity (Figure 3 C). The bushbaby protein is shorter than the human and gorilla versions, as it misses a part of the low complexity region, but the region that was aligned with the chemokine structure (indicated with ‘c’ as putative chemokine domain in Figure 3 C) is well conserved in all orthologs. Also the RGD-motif is well conserved in all species (orange box in Figure 3 C). The N-Glycosylation site in LCR is missing in bushbaby but moved to the position of a cysteine (highlighted in pink in Figure 3C) that is located in first beta strand and does not form a disulfide bond in the chemokine-like model of N73.

The N73 gene (Gene ID: ENSG00000184334 in Ensembl release 61) comprises 3 exons like many other chemokines; however, the protein coding region lies only on the first one. The open reading frame is located on chromosome 8q24.3. At present, no other chemokines are known to be located on this chromosome. Close to the N73 gene, an orphan G protein coupled receptor (GPR20) is found (Figure S4 B). The annotation of the human N73 gene produced by the automatic Ensembl *genebuild* prediction method did not detect the N73 gene in more recent Ensembl releases (62 and higher) for reasons not specified on the website, although the genomic sequence is identical to the sequence of previous versions. Interestingly, the orthologous gorilla and bushbaby genes of N73 are still annotated as novel protein coding genes in the current Ensembl release 64 (September 2011).

Furthermore, analysis of the promoter region of N73 for transcription factor binding sites identified several binding sites (Supporting Information Table S6), which are known from other chemokines like NF-IL6, NF-kappaB and AP1(C-Jun) .

Supporting Materials and Methods

Consensus signal peptide prediction

Signal peptides (SP) were predicted and removed by using the consensus of two different prediction methods: PrediSi and SigPfam . PrediSi has been trained with datasets consisting of 2,783 protein sequences from eukaryotes, 557 sequences from Gram-negative bacteria and 236 sequences from Gram-positive bacteria . SigPfam has been trained with 270 Human sequences . We tested both methods using default parameters and a dataset of protein sequences with and without annotated signal peptides. Each test set (positive and negative) contained 24 protein sequences (12 eukaryotic and 12 prokaryotic: see below Dataset S1). Based on the annotated positions of the signal peptides in the test set, we used the scores calculated by each method to assign confidence groups: VHC: very high confidence HC: high confidence, MC: medium confidence, LC: low confidence, VLC: very low confidence. Then, we derived a consensus prediction value by combining the individual predictions and confidence groups in order to assess the prediction power of each method and to establish rules to be applied to the query sequences:

1.The cleavage site prediction is considered negative in case the SP length is > 70.

2. High (HC) and low (LC) confidence groups for positive (‘+’) and negative (‘-’) signal peptide predictions were assigned to the scores given by the 2 methods:

- PrediSi: HC^+^: ≥0.8, LC^+^: <0.8-0.5, LC^-^: <0.5-0.4, HC^-^: <0.4

- SigPfam: HC^+^: ≤0.0095, LC^+^: >0.0095-0.2, LC^-^: >0.20-0.7, HC^-^: >0.7

3. Confidence groups from each method were combined to obtain the final prediction: 2xHC^+^ = VHC^+^; HC^+^ & LC^+^ = HC^+^ (if there was cleavage site agreement), otherwise MC^+^; 2xLC^+^ = MC^+^ (if there was cleavage site agreement), otherwise LC^+^; 2xLC^-^ = LC^-^; HC^-^ & LC^-^ = HC^-^; 2xHC^-^ = VHC^-^; all other combinations = contradictive (VLC).

4. Predicted signal peptides were removed from the query sequences, if they were classified as VHC^+^, HC^+^ or MC^+^ and the predicted cleavage site agreed ±2 residues.

**Dataset S1:** Signal peptide test dataset (SwissProt IDs):

- **Signal peptide positive Eukaryota:** 5NTD_HUMAN, EMP47_YEAST, 1A01_PONPY, 1A02_HUMAN, 2SS_BEREX, 5NTD_RAT, 11S3_HELAN, AGLU_CANTS, PEPAF_RABIT, PEPA_PIG, LYAM3_HUMAN, FLRT2_HUMAN,
- **Signal peptide positive Prokaryota:** FHUE_ECOLI, AMPC_SERMA, PORD_PSEAE, MSME_STRMU, OMPH_PHOPR, AMPC_CITFR, AMPC_ECOLI, DEXT_STRDO, AMPC_ENTCL, FPTA_PSEAE, LPPL_PSEAE, ASA1_ENTFA,
- **Signal peptide negative Eukaryota:** ELL_MOUSE, GLRX_RABIT, HMTI_DROME, PHO2_YEAST, DEFA_ANTMA, ATN1_BUFMA, CGD1_HUMAN, SM22_CHICK, DLX6_BRARE, DRG1_RAT, C6A1_MUSDO, MT3_CARPA,
- **Signal peptide negative Prokaryota:** FIXI_RHIME, FTH2_HAEIN, YQKF_BACSU, ALAM_RHOER, GCTA_ACIFE, YEAW_ECOLI, CATA_ACICA, CLCA_RHOOP, ODH_ARTSP, FTSH_MYCPN, LEU1_LACLA, DYR_CITFR

Consensus transmembrane prediction

Transmembrane (TM) regions were predicted and removed by using the consensus of 2 different prediction methods: TMHMM and Memsat . TMHMM has been trained with a dataset consisting of 160 transmembrane proteins from prokaryotes and eukaryotes that are either single- or multi-spanning . Memsat has been trained with datasets consisting of 83 integral membrane proteins taken from prokaryotes and eukaryotes . We tested both methods using default parameters and datasets of protein sequences with and without annotated transmembrane regions. It is important to notice that the assigned confidence groups for the consensus prediction are based on the predicted length of the transmembrane region and not on the scores as for the signal peptides. It is known that the average length of a TM helix is 21 residues, and 94% of all TM segments have a length between 17 and 25 residues . According to the TM segment length, we assigned confidence groups for each predicted membrane segment: High confidence (HC)= predicted TM length 15-27 residues; Medium confidence (MC)= predicted TM length 8-14 and 28-31 residues; Low confidence (LC)= predicted TM length 2-7 residues. In case the segment length corresponded to HC and MC predictions (length 8-31 residues), the predicted TM region was removed from the query sequence for further analysis.

References

1. Letunic I, Doerks T, Bork P (2009) SMART 6: recent updates and new developments. Nucleic Acids Res 37: D229-232.

2. Schultz J, Milpetz F, Bork P, Ponting CP (1998) SMART, a simple modular architecture research tool: identification of signaling domains. Proc Natl Acad Sci U S A 95: 5857-5864.

3. Bateman A, Coin L, Durbin R, Finn RD, Hollich V, et al. (2004) The Pfam protein families database. Nucleic Acids Res 32: D138-141.

4. Sigrist CJ, Cerutti L, Hulo N, Gattiker A, Falquet L, et al. (2002) PROSITE: a documented database using patterns and profiles as motif descriptors. Brief Bioinform 3: 265-274.

5. Lata S, Raghava GP (2009) Prediction and classification of chemokines and their receptors. Protein Eng Des Sel 22: 441-444.

6. Luttichau HR, Johnsen AH, Jurlander J, Rosenkilde MM, Schwartz TW (2007) Kaposi sarcoma-associated herpes virus targets the lymphotactin receptor with both a broad spectrum antagonist vCCL2 and a highly selective and potent agonist vCCL3. J Biol Chem 282: 17794-17805.

7. Allen SJ, Crown SE, Handel TM (2007) Chemokine: receptor structure, interactions, and antagonism. Annu Rev Immunol 25: 787-820.

8. Pease JE, Williams TJ (2006) The attraction of chemokines as a target for specific anti-inflammatory therapy. Br J Pharmacol 147 Suppl 1: S212-221.

9. Hoover DM, Boulegue C, Yang D, Oppenheim JJ, Tucker K, et al. (2002) The structure of human macrophage inflammatory protein-3alpha /CCL20. Linking antimicrobial and CC chemokine receptor-6-binding activities with human beta-defensins. J Biol Chem 277: 37647-37654.

10. Pisabarro MT, Leung B, Kwong M, Corpuz R, Frantz GD, et al. (2006) Cutting edge: novel human dendritic cell- and monocyte-attracting chemokine-like protein identified by fold recognition methods. J Immunol 176: 2069-2073.

11. Horton P, Park KJ, Obayashi T, Fujita N, Harada H, et al. (2007) WoLF PSORT: protein localization predictor. Nucleic Acids Res 35: W585-587.

12. Assa-Munt N, Jia X, Laakkonen P, Ruoslahti E (2001) Solution structures and integrin binding activities of an RGD peptide with two isomers. Biochemistry 40: 2373-2378.

13. Frisch SM, Screaton RA (2001) Anoikis mechanisms. Curr Opin Cell Biol 13: 555-562.

14. Kim JE, Kim SJ, Jeong HW, Lee BH, Choi JY, et al. (2003) RGD peptides released from beta ig-h3, a TGF-beta-induced cell-adhesive molecule, mediate apoptosis. Oncogene 22: 2045-2053.

15. Chong IW, Shi MM, Love JA, Christiani DC, Paulauskis JD (2000) Regulation of chemokine mRNA expression in a rat model of vanadium-induced pulmonary inflammation. Inflammation 24: 505-517.

16. Gerber A, Heimburg A, Reisenauer A, Wille A, Welte T, et al. (2004) Proteasome inhibitors modulate chemokine production in lung epithelial and monocytic cells. Eur Respir J 24: 40-48.

17. Ueda Y, Su Y, Richmond A (2007) CCAAT displacement protein regulates nuclear factor-kappa beta-mediated chemokine transcription in melanoma cells. Melanoma Res 17: 91-103.

18. Widmer U, Manogue KR, Cerami A, Sherry B (1993) Genomic cloning and promoter analysis of macrophage inflammatory protein (MIP)-2, MIP-1 alpha, and MIP-1 beta, members of the chemokine superfamily of proinflammatory cytokines. J Immunol 150: 4996-5012.

19. Genin P, Algarte M, Roof P, Lin R, Hiscott J (2000) Regulation of RANTES chemokine gene expression requires cooperativity between NF-kappa B and IFN-regulatory factor transcription factors. J Immunol 164: 5352-5361.

20. Roebuck KA (1999) Regulation of interleukin-8 gene expression. J Interferon Cytokine Res 19: 429-438.

21. Yeruva S, Ramadori G, Raddatz D (2008) NF-kappaB-dependent synergistic regulation of CXCL10 gene expression by IL-1beta and IFN-gamma in human intestinal epithelial cell lines. Int J Colorectal Dis 23: 305-317.

22. Hiller K, Grote A, Scheer M, Munch R, Jahn D (2004) PrediSi: prediction of signal peptides and their cleavage positions. Nucleic Acids Res 32: W375-379.

23. Zhang Z, Henzel WJ (2004) Signal peptide prediction based on analysis of experimentally verified cleavage sites. Protein Sci 13: 2819-2824.

24. Sonnhammer EL, von Heijne G, Krogh A (1998) A hidden Markov model for predicting transmembrane helices in protein sequences. Proc Int Conf Intell Syst Mol Biol 6: 175-182.

25. Jones DT, Taylor WR, Thornton JM (1994) A model recognition approach to the prediction of all-helical membrane protein structure and topology. Biochemistry 33: 3038-3049.

26. Pasquier C, Promponas VJ, Palaios GA, Hamodrakas JS, Hamodrakas SJ (1999) A novel method for predicting transmembrane segments in proteins based on a statistical analysis of the SwissProt database: the PRED-TMR algorithm. Protein Eng 12: 381-385.
